# Comparison of strand-specific transcriptomes of enterohemorrhagic *Escherichia coli* O157:H7 EDL933 (EHEC) under eleven different environmental conditions including radish sprouts and cattle feces

**DOI:** 10.1186/1471-2164-15-353

**Published:** 2014-05-09

**Authors:** Richard Landstorfer, Svenja Simon, Steffen Schober, Daniel Keim, Siegfried Scherer, Klaus Neuhaus

**Affiliations:** Lehrstuhl für Mikrobielle Ökologie, Wissenschaftszentrum Weihenstephan, Technische Universität München, Weihenstephaner Berg 3, D-85350 Freising, Germany; Lehrstuhl für Datenanalyse und Visualisierung, Fachbereich Informatik und Informationswissenschaft, Universität Konstanz, Box 78, D-78457 Konstanz, Germany; Institut für Nachrichtentechnik, Universität Ulm, Albert-Einstein-Allee 43, D-89081 Ulm, Germany

**Keywords:** EHEC, Radish sprouts, Cattle feces, Environment, Global transcriptome, Next Generation Sequencing, SOLiD-Illumina comparison, Background transcription, Hypothetical genes

## Abstract

**Background:**

Multiple infection sources for enterohemorrhagic *Escherichia coli* O157:H7 (EHEC) are known, including animal products, fruit and vegetables. The ecology of this pathogen outside its human host is largely unknown and one third of its annotated genes are still hypothetical. To identify genetic determinants expressed under a variety of environmental factors, we applied strand-specific RNA-sequencing, comparing the SOLiD and Illumina systems.

**Results:**

Transcriptomes of EHEC were sequenced under 11 different biotic and abiotic conditions: LB medium at pH4, pH7, pH9, or at 15°C; LB with nitrite or trimethoprim-sulfamethoxazole; LB-agar surface, M9 minimal medium, spinach leaf juice, surface of living radish sprouts, and cattle feces. Of 5379 annotated genes in strain EDL933 (genome and plasmid), a surprising minority of only 144 had null sequencing reads under all conditions. We therefore developed a statistical method to distinguish weakly transcribed genes from background transcription. We find that 96% of all genes and 91.5% of the hypothetical genes exhibit a significant transcriptional signal under at least one condition. Comparing SOLiD and Illumina systems, we find a high correlation between both approaches for fold-changes of the induced or repressed genes. The pathogenicity island LEE showed highest transcriptional activity in LB medium, minimal medium, and after treatment with antibiotics. Unique sets of genes, including many hypothetical genes, are highly up-regulated on radish sprouts, cattle feces, or in the presence of antibiotics. Furthermore, we observed induction of the shiga-toxin carrying phages by antibiotics and confirmed active biofilm related genes on radish sprouts, in cattle feces, and on agar plates.

**Conclusions:**

Since only a minority of genes (2.7%) were not active under any condition tested (null reads), we suggest that the assumption of significant genome over-annotations is wrong. Environmental transcriptomics uncovered hitherto unknown gene functions and unique regulatory patterns in EHEC. For instance, the environmental function of *azoR* had been elusive, but this gene is highly active on radish sprouts. Thus, NGS-transcriptomics is an appropriate technique to propose new roles of hypothetical genes and to guide future research.

**Electronic supplementary material:**

The online version of this article (doi:10.1186/1471-2164-15-353) contains supplementary material, which is available to authorized users.

## Background

Humans infected by enterohemorrhagic *Escherichia coli* O157:H7 (EHEC) suffer from gastroenteritis. Sometimes they develop hemorrhagic colitis or hemolytic uremic syndrome which can cause kidney failure [1, 2]. Treatment of an EHEC infection with antibiotics is under debate since this can increase the risk for the hemolytic uremic syndrome [3]. Therefore, much effort should be put into prevention of transmission. However, this is complicated due to the low infectious dose of less than 50 bacterial cells [4]. Infection sources are multiple [5, 6]: bacteria can persist and reproduce in soil, dung, water or other environmental niches, eventually causing fresh produce to be contaminated [7]. Typical vectors for EHEC outbreaks include spinach, apple juice, unpasteurized milk, lettuce, but also meat products such as sausage [2]. A large outbreak in Japan 1996 caused more than 6000 infections and was due to contaminated radish sprouts [8]. Fenugreek sprouts (*Trigonella foenum-graecum*) caused a severe outbreak with more than 3800 infected and 53 dead in Germany in 2011. The sprouts were contaminated with a related bacterium, *Escherichia coli* O104:H4 [9, 10]. Thus, the spectrum of environmental niches of pathogenic *E. coli* is quite large, ranging from water, single cell organisms to plant and lower animals and vertebrates [7, 11, 12].

Gene regulation of EHEC has been studied under individual conditions using microarrays or related techniques [13–15]. However, microarrays are limited, especially when examining rare or highly abundant transcripts or unknown genes. New methods in transcriptome analysis such as strand-specific RNA-seq using Next Generation Sequencing (NGS) technologies have a much higher resolution [16]. To date, only a few studies examined bacterial pathogens (e.g. [17–19]). In this work, we applied strand-specific RNA-seq to EHEC to identify genes involved in environmental and plant persistence with a special focus on hypothetical genes.

About one third of the genes of EHEC are still annotated as hypothetical. Hypothetical proteins are defined as genes that have no homology to any other predicted protein in any species [20] and the function of these genes is largely unknown. After sequencing a new genome their existence is predicted by annotation tools, e.g., GLIMMER [20, 21] or GeneMarkS [22]. At this stage, there is no experimental evidence for the expression of these genes. A characterization of all hypothetical genes at the current rate would take decades [23, 24]. However, transcription studies allow confirmation of the activity of hypothetical genes, pre-characterize and remove them from the hypothetical category [24, 25]. The expression of some hypothetical proteins of EHEC has already been reported in single environmental studies, e. g., during heat shock [26] or in adhesion to bovine epithelial cells [27]. However, global approaches, which cover a large environmental spectrum to identify functional hypothetical genes, are still missing. We therefore sequenced the transcriptomes of several EHEC-cultures from a high diversity of conditions strand-specifically to derive transcriptional patterns and global trends.

## Results and discussion

### Sequencing statistics

**Figure 1:**
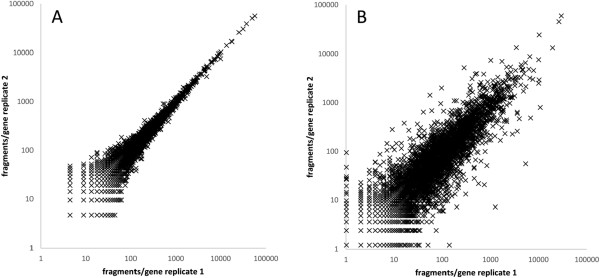
**Comparison of technical and biological replicates. A:** The technical replicate was generated from spinach medium by splitting the libraries before PCR of SOLiD sequencing. The mapped counts were normalized and are given in fragments/gene according to Haas *et al.* (51). The correlation coefficient *R* ^2^ is virtually 1.0. **B:** Biological replicates for LB medium are shown. They were sequenced on two different platforms, the SOLiD 4.0 system and the Illumina MiSeq sequencer, to exclude technical artifacts of one platform. The correlation coefficient *R* ^2^ is 0.72.

In order to test the reproducibility of the sequencing process, two technical replicates of barcoded libraries of two conditions were generated, spinach medium and LB-nitrite. After cDNA-synthesis the libraries were split and treated independently and the RPKM values of each replicate were compared. The correlation coefficient *R*^2^ was analyzed as described in Haas *et al.*[28] in reads per gene. Since the correlation was excellent (*R*^2^ = 1.0, see Figure 1A), as had also been observed for other NGS experiments (e.g., [29]), we combined those technical replicates for further expression analysis. Next, biological reproducibility was tested by sequencing replicates of the LB reference and the radish sprout condition on two different sequencing platforms SOLiD and Illumina. Despite massive differences in library making techniques and in the sequencing strategy of both platforms, we obtained a high correlation of *R*^2^ = 0.72 (Figure 1B). This verified that the observed changes in gene regulation were not due to technical or experimental artifacts.

Taking all sequencing results together, 26.1 million high quality reads mapped to the EHEC genome and to the plasmid pO157 (see Table 1 for a summary of the sequencing statistics). Since total RNA contains up to 95% rRNA [30], this RNA species was depleted before sequencing. However, averaged over all conditions, 26.4% of the sequenced RNA is remaining rRNA (Table 1). About 1.4% of all reads mapped to the plasmid (Table 1). The plasmid is 92,077 bp in length, which is about 1.7% of the 5,528,445 bp genome. Assuming a comparable transcription of genome and plasmid encoded genes, we calculate the number of plasmid pO157 in a single bacterial cell to be in parity with the genome.

**Table 1:** **Sequencing statistics for the eleven conditions** ^**1**^

Condition	Mappable reads			rRNA	RPKM background transcription	Significant active genes (p ≤ 0.05)	% hypotheticals in active genes	Significant active hypotheticals (p ≤ 0.05)
	Genome	Plasmid	Total					
**LB (SOLiD)**	1,990,326	46,497	2,036,823	13.0%	0.2	4,557	29.7%	1355
**LB (Illumina)**	3,301,130	5,215	3,306,345	25.2%	0.5	4,463	29.4%	1313
**LB-pH9**	2,953,471	40,607	2,994,078	22.3%	0.1	4,445	29.4%	1308
**LB-pH4**	1,315,629	5,353	1,320,982	7.3%	0.03	3,441	27.0%	930
**LB-15°C**	2,251,249	42,964	2,294,213	11.3%	0.1	4,360	29.7%	1292
**LB-nitrite**	2,143,433	48,449	2,191,882	29.7%	0.2	4,377	29.2%	1277
**LB-antibiotics**	718,712	3,615	722,327	14.0%	0.03	2,892	27.1%	785
**LB-solid**	1,679,329	18,652	1,697,981	20.0%	0.02	4,006	28.7%	1150
**minimal medium**	1,496,231	34,135	1,530,366	25.4%	0.1	4,017	28.7%	1151
**spinach juice**	1,638,842	71,874	1,710,716	8.8%	0.08	4,131	29.4%	1215
**radish sprouts (SOLiD)**	1,355,143	9,876	1,365,019	38.1%	0.09	4,079	28.9%	1180
**radish sprouts (Illumina)**	3,724,713	31,160	3,755,873	60.3%	0.29	3,852	26.8%	1035
**feces**	1,184,557	4,457	1,189,014	30.5%	0.05	2,979	28.9%	861
**total**	25,752,765	362,854	26,115,619	26.4%	Ø 0.14	5,142	26.5%	1621

^1^The number of mappable reads within each condition is listed for the genome and plasmid. The percentage of remaining rRNA is shown below.

### Background transcription and silent genes

Random transcription, also called background transcription or transcriptional noise, has been reported in NGS studies of prokaryotes and eukaryotes (e.g., [31–33]). Single reads distribute all over the genome and are found in coding regions, non-coding regions and antisense to annotated genes. Most of such reads apparently do not form transcriptional units, i.e. they do not originate from non-annotated genes for most cases. It is unclear whether the reads occur due to background noise introduced during deep sequencing experiments or whether they are caused by the low information content of bacterial promoters, resulting in “sloppy” transcription [34, 35]. To see whether such reads mapped simply by chance to the EHEC genome, the RNA-seq data of LB medium in this study was mapped to the mouse Y-chromosome (95 Mbp). Out of 7 million reads, only one matched to the mouse genome sequence, thus all reads appear to be specific.

Generally, transcriptional noise is disregarded as non-functional. However, background transcription interferes with the detection of weakly transcribed genes. Several attempts were made to estimate a threshold to consider a gene as being active. Filiatrault *et al.*[18] used proteomics data to estimate a threshold for an active gene in comparison with RNA-seq. Mortazavi *et al.*[29] already estimated an upper bound of background noise in mouse transcriptomes by estimating the RPKM of all regions outside of exons or other transcribed regions, but this inevitably includes non-annotated genes causing a higher upper bound. However, mostly cut-off values have been selected intuitively. For instance, Beaume *et al.*[36] defined a gene as being significantly active if its transcription is higher than 0.5 of the average sequencing coverage. The disadvantage of all methods applied hitherto is that weakly transcribed genes are below the threshold.

To detect weakly transcribed genes an estimate of a threshold of background transcription was performed for EHEC in order to define a gene as being active. To derive such a threshold value, the background transcription level under different conditions was observed using manually selected regions of the genome which are devoid of annotated genes and any conspicuous transcriptional patterns. These regions comprise a total of 104,192 bp or about 2% of the genome (see Methods). Table 1 lists the RPKM values of background transcription for each condition. The average RPKM value for all conditions, including the biological replicates, is 0.14 (±0.13, standard deviation). In order to see if the “RPKM of the background transcription” is dependent on the sequencing technology used (Illumina or SOLiD), we analyzed an additional data set from EHEC prepared according to the Illumina technology (data not shown). The average background RPKM of 0.13 was found to be in a similar range compared to the eleven conditions sequenced with the SOLiD technology. Thus, the mean level of background transcription compares to a 750 bp stretch of DNA covered by one read in a sequenced library of 10 million reads in EHEC.

For each gene, the probability whether its reads result from background or from activity above background, was calculated (see Additional file 1: Table S2). Of 5,379 annotated genes, the activity of 5,142 was found to be significantly above background (p ≤ 0.05), thus they do not originate from the noise (Table 1).

**Figure 2:**
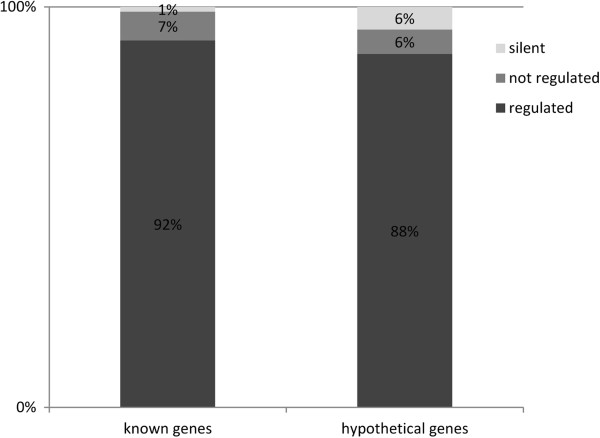
**Percentage of regulated hypothetical and known genes.** The category of hypothetical genes includes all genes that are annotated as hypothetical protein (1771 genes). All other genes are included in the category of known genes (3608 genes). We consider a gene as regulated if its logFC is ≥ 3 or ≤ −1 in at least one condition. A gene is silent if the RPKMs in all conditions are below the threshold for random transcription. Consequently, silent genes are also not regulated.

Filtering for transcriptionally inactive genes at any of the conditions studied, we found only 144 inactive genes which is about 2.7% of the annotated genes (Additional file 2: Table S3). These genes are covered by no read under any of the conditions investigated. 69.4% of the silent genes are hypothetical genes, indicating a potential over-annotation. On the other hand, some hypothetical genes might only be active at conditions not yet probed.We considered a gene as being regulated if its logFC was ≥ 3 or ≤ −1 under at least one condition. Accordingly, the number of regulated genes is about 4% higher for the known genes compared to the hypothetical genes (Figure 2).

### Overall comparison of transcriptomes

**Figure 3:**
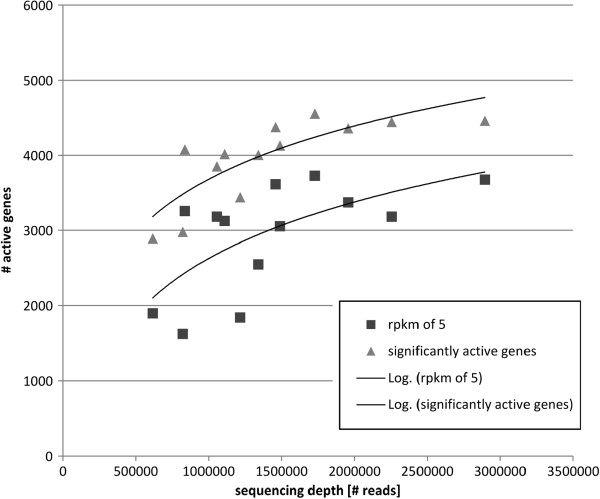
**Correlation of sequencing depth and number of active genes.** Active genes are defined as genes with a probability ≤ 0.05 to originate from background transcription. Additionally, the number of active genes is shown with an RPKM of 5 (about 40 × average random RPKM). An averaged correlation for each data set is shown using a logarithmic trend line.

It was observed that the number of active genes differs for different conditions (Table 1). In feces, the number of active genes is more than 1,000 genes lower compared to sprouts, although both conditions have about the same sequencing depth. This is important since differences in numbers of active genes could have originated from different sequencing depths as this influences the chance of finding a transcript. We show that such an effect of the sequencing depth does indeed influence the number of genes which will be defined as active (Figure 3): the number of active genes asymptotically reaches saturation with an increase in sequencing depth. The same pattern was observed by Haas *et al.*[28], also for EHEC EDL933. Vivancos *et al.*[33] show a similar effect for RNA-seq in *Mycoplasma pneumonia* and *Mus musculus*. However, the sequencing depth for EHEC grown on radish sprouts and feces is about the same. Therefore, the major difference observed must be of biological significance. We assume that survival of EHEC on radish sprouts requires a larger number of active genes than persistence in cattle feces since the cells have to deal with many environmental factors such as differing water activities, osmotic stress, radiation, temperature changes and low nutrient contents which are not present in cattle feces.

**Figure 4:**
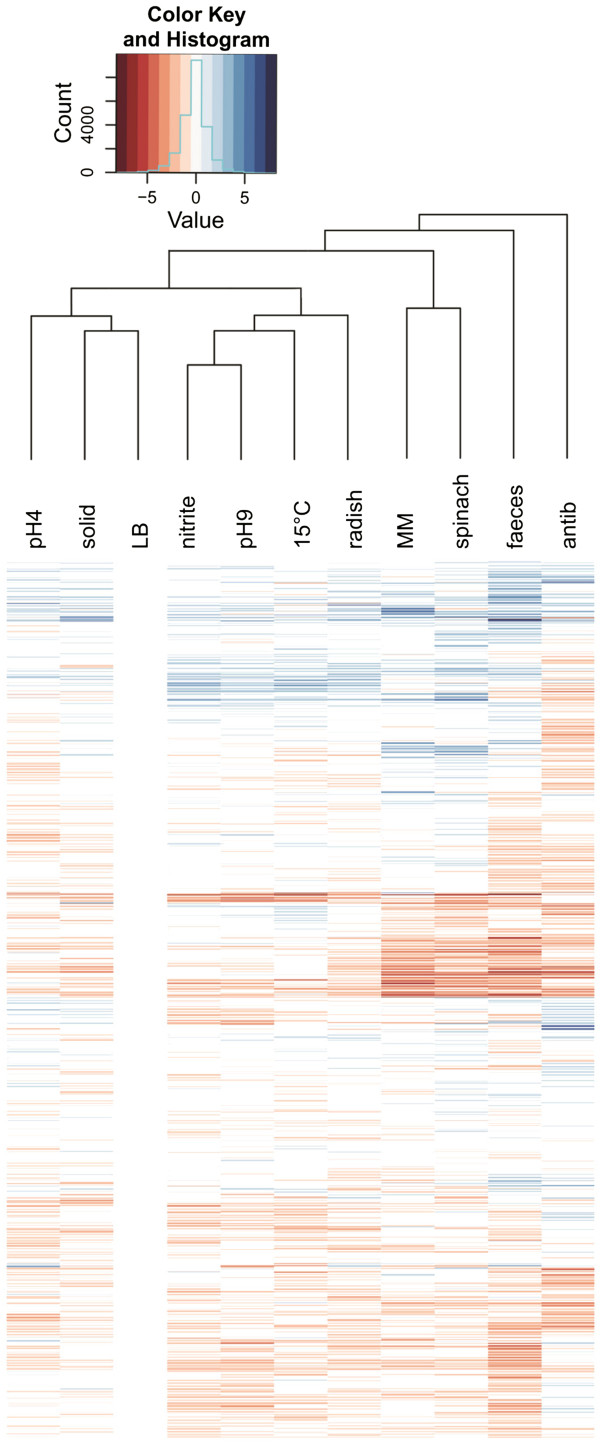
**Heat map representing 2026 regulated genes.** Only those genes are displayed that are covered by reads in all of the conditions sequenced on the SOLiD system. Genes are clustered for similar regulation patterns among the conditions (vertical columns). Each horizontal column represents a different condition. On the right a similarity tree for the conditions is shown. The heat map was calculated on the relative differences (logFCs) in transcription levels to the reference condition LB. Transcription values higher than in LB are shown in shades of blue, transcription values lower are shown in shades of red. LB, lysogeny broth; pH9, LB-pH9; nitrite, LB-nitrite; spinach, spinach medium; radish, radish shoots; MM, minimal medium; antib, LB-antibiotics; solid, LB-solid; pH4, LB-pH4; faeces, cattle feces; 15°C, LB-15°C.

With only 2892 active genes, LB-antibiotics has the lowest number of active genes of all. In comparison, the reference condition LB displays around 4500 active genes (Table 1). Admittedly, LB-antibiotics has the lowest sequencing depth of all. However, as can be seen from Figure 3, the number of active genes is disproportionately low. After antibiotic treatment the cells elongate several times their original cell length. The indirect block of DNA synthesis influences their regulational pattern. Genes of many different pathways are turned off. We visualized this transcriptional pattern of LB-antibiotics in the heat map distance tree (Figure 4). The up-regulated genes (colored in blue) and down-regulated genes (colored in red) do not form the regulatory clusters observed in the other ten conditions: LB-antibiotics forms an outer group (antib in Figure 4). The extreme stress leads to most severe transcriptomic differences. Interestingly, it is the only LB-condition not clustering together with the other LB-based experiments. The four conditions that do not originate from LB medium, i.e. spinach medium, minimal medium, and feces, show a more related regulational pattern. Radish sprouts are closer to the conditions which originated from LB medium. We assume the high similarity of minimal medium and spinach medium as being due to a low nutrient content in both conditions. LB-pH9 and LB-nitrite have the most similar transcriptomic pattern, despite LB-nitrite being slightly acidic (Figure 4).

### Transcriptional activity of hypothetical genes

We examined the transcriptional regulation of the 5379 protein-coding genes (GenBank and RefSeq) for the genome and plasmid in EHEC (Additional file 3: Table S4). Out of these genes, 2266 are not in COGs (cluster of orthologous genes), have a general function prediction only or are annotated as hypothetical (completely unknown function). Of the annotated genes on the genome, 32.9% are hypothetical. Table 1 shows a summary of active hypotheticals for each condition. In total, 77.0% of them are active in at least one condition (Table 1). Formerly, most experiments using *E. coli* refer to standard LB at pH7 or minimal medium. We hypothesized to find additional uniquely up-regulated hypothetical genes under non-standard laboratory conditions. Concentrating on highly regulated genes by using very stringent cut-off thresholds only (logFCs ≥ 5 at a single condition), we found 26 hypothetical genes in LB with antibiotics, 14 in minimal medium, 13 in feces, nine on radish sprouts, and nine in spinach medium. In contrast, three hypothetical genes are active in LB at 15°C, three in LB at pH4, two on solid LB, one in LB with nitrite, and none on LB at pH9 (Table 2, graphic version in Additional file 4). We performed a BLAST search (blastp) to evaluate the taxonomic distribution of these genes. Hits with an E value threshold of 10^−5^ or lower were taken as indicator for the maximal taxonomic distribution of this gene. According to this definition, 35 hypothetical genes are present only within the genus *Escherichia*, 17 within Enterobacteriaceae, 19 within proteobacteria, 7 within bacteria, and 2 within “cellular organisms”, respectively.

**Table 2:** **Hypothetical genes with a logFC ≥ 5 in transcription levels in a single condition compared to LB** ^**1**^

Gene tag	Product	Ref’s	gene active in	LB	LB-pH9	LB-pH4	LB-15°C	LB-nitrite	LB-antibiotics	LB-solid	minimal medium	spinach juice	radish sprouts	feces
Z0840	hypothetical protein		LB-pH4	1 (2)	1.4 (3)	**5.5 (18)**	2.0 (8)	−1.4 (0)	−1.4 (0)	−1.4 (0)	−1.4 (0)	2.9 (10)	−1.4 (0)	**4.4 (9)**
Z1576	hypothetical protein		LB-pH4	1 (0)	0.0 (0)	**5.3 (3)**	**3.4 (4)**	0.0 (0)	0.0 (0)	0.0 (0)	0.0 (0)	0.0 (0)	3.0 (2)	0.0 (0)
Z1850	hypothetical protein		LB-pH4	1 (0)	0.0 (0)	**5.3 (3)**	0.0 (0)	0.0 (0)	0.0 (0)	0.0 (0)	0.0 (0)	0.0 (0)	0.0 (0)	0.0 (0)
Z4062	hypothetical protein		LB-15°C	1 (9)	**2.7 (36)**	−4.4 (0)	**5.1 (275)**	1.5 (26)	−4.4 (0)	1.3 (8)	−4.4 (0)	0.4 (7)	0.1 (5)	−4.4 (0)
Z4925	hypothetical protein		LB-15°C	1 (114)	**2.4 (388)**	0.2 (23)	**5.0 (3365)**	**3.3 (1157)**	**−9.0 (0)**	−1.1 (18)	**2.7 (482)**	**4.1 (1163)**	**1.6 (210)**	−0.1 (18)
Z5688	hypothetical protein		LB-15°C	1 (2)	1.7 (5)	−3.6 (0)	**5.5 (90)**	**2.2 (10)**	−3.6 (0)	−3.6 (0)	0.0 (1)	1.2 (3)	**2.2 (6)**	−3.6 (0)
Z1924	hypothetical protein		LB-nitrite	1 (9)	**5.3 (250)**	**3.7 (23)**	**4.3 (182)**	**6.0 (659)**	−3.9 (0)	**4.7 (90)**	**3.2 (59)**	1.6 (18)	**4.1 (101)**	**4.7 (47)**
Z0314	prophage CP-933H, tail fiber		LB-antibiotics	1 (5)	−0.3 (2)	0.4 (1)	0.4 (6)	−0.7 (3)	**6.0 (92)**	−0.7 (1)	−0.3 (3)	−0.6 (2)	**−4.9 (0)**	−4.9 (0)
Z0316	prophage CP-933H, tail fiber		LB-antibiotics	1 (12)	−0.6 (4)	−0.5 (1)	−0.1 (9)	0.6 (17)	**5.3 (123)**	0.4 (5)	−1.2 (3)	−0.6 (5)	**−2.8 (1)**	0.1 (2)
Z0344	hypothetical protein		LB-antibiotics	1 (1)	1.3 (2)	3.9 (3)	1.1 (2)	2.2 (5)	**5.2 (12)**	−1.4 (0)	−1.4 (0)	2.8 (5)	1.6 (2)	−1.4 (0)
Z0392	hypothetical protein		LB-antibiotics	1 (0)	2.8 (1)	0.0 (0)	0.0 (0)	0.0 (0)	**5.6 (3)**	0.0 (0)	**3.6 (2)**	0.0 (0)	0.0 (0)	0.0 (0)
Z0949	prophage CP-933K	[37]	LB-antibiotics	1 (17)	0.5 (14)	**2.4 (15)**	0.2 (17)	−0.7 (10)	**5.0 (143)**	−4.9 (0)	0.3 (13)	−1.5 (3)	0.3 (12)	**2.6 (17)**
Z1098	hypothetical protein		LB-antibiotics	1 (1)	−2.1 (0)	−2.1 (0)	1.8 (3)	1.5 (3)	**5.1 (11)**	−2.1 (0)	1.5 (2)	−2.1 (0)	0.9 (1)	−2.1 (0)
Z1433	prophage BP-933W	[37]	LB-antibiotics	1 (15)	0.5 (13)	−5.1 (0)	0.8 (22)	1.0 (29)	**5.9 (231)**	−5.1 (0)	0.0 (10)	−0.8 (5)	0.3 (11)	0.8 (4)
Z1434	prophage BP-933W	[37, 38]	LB-antibiotics	1 (5)	2.2 (14)	2.4 (4)	**2.3 (21)**	2.2 (21)	**7.2 (203)**	1.3 (4)	**2.6 (19)**	0.4 (3)	1.6 (8)	−3.0 (0)
Z1441	prophage BP-933W	[37]	LB-antibiotics	1 (274)	0.0 (167)	−0.3 (35)	1.4 (607)	0.9 (464)	**5.5 (3123)**	−0.8 (52)	1.3 (424)	0.0 (160)	**1.6 (482)**	**−2.5 (8)**
Z1501	hypothetical protein		LB-antibiotics	1 (27)	−0.1 (15)	−0.3 (4)	0.0 (23)	−0.1 (24)	**5.1 (235)**	−0.1 (8)	0.1 (18)	−0.7 (10)	1.3 (38)	−0.7 (3)
Z1656	hypothetical protein		LB-antibiotics	1 (0)	−1.4 (0)	−1.4 (0)	−1.4 (0)	−1.4 (0)	**5.2 (7)**	−1.4 (0)	−1.4 (0)	−1.4 (0)	1.6 (1)	−1.4 (0)
Z1840	hypothetical protein		LB-antibiotics	1 (0)	0.0 (0)	0.0 (0)	0.0 (0)	2.7 (4)	**5.6 (9)**	0.0 (0)	0.0 (0)	3.3 (4)	0.0 (0)	0.0 (0)
Z3353	hypothetical protein		LB-antibiotics	1 (0)	0.0 (0)	0.0 (0)	0.0 (0)	0.0 (0)	**7.9 (29)**	0.0 (0)	0.0 (0)	0.0 (0)	3.0 (2)	0.0 (0)
Z3369	prophage CP-933V	[37]	LB-antibiotics	1 (7)	−0.3 (3)	−3.9 (0)	0.4 (7)	−1.2 (2)	**5.7 (92)**	−3.9 (0)	−0.3 (3)	−3.9 (0)	1.9 (15)	−3.9 (0)
Z3370	prophage CP-933V	[37]	LB-antibiotics	1 (32)	**−2.6 (3)**	−2.4 (1)	−0.3 (21)	−1.7 (9)	**5.4 (312)**	−1.3 (4)	**−2.2 (4)**	**−4.4 (1)**	0.7 (30)	**−7.8 (0)**
Z3371	prophage CP-933V	[37]	LB-antibiotics	1 (6)	**2.0 (16)**	−4.2 (0)	**2.9 (42)**	1.3 (15)	**7.6 (337)**	1.0 (4)	−4.2 (0)	−0.9 (2)	1.2 (9)	−4.2 (0)
Z3372	prophage CP-933V		LB-antibiotics	1 (14)	1.4 (22)	−5.3 (0)	0.6 (18)	0.0 (13)	**5.1 (126)**	−5.3 (0)	−0.8 (5)	0.9 (15)	0.7 (13)	0.5 (3)
Z3609	restricted to *Escherichia*		LB-antibiotics	1 (3)	1.2 (4)	−4.7 (0)	1.1 (6)	0.2 (3)	**5.4 (35)**	−4.7 (0)	−4.7 (0)	0.1 (2)	**−4.7 (0)**	**3.1 (5)**
Z4174	hypothetical protein		LB-antibiotics	1 (2)	2.1 (6)	−2.1 (0)	**4.2 (37)**	**3.8 (31)**	**5.5 (30)**	**3.1 (7)**	1.5 (4)	**2.7 (9)**	0.9 (2)	−2.1 (0)
Z4183	hypothetical protein		LB-antibiotics	1 (0)	0.0 (0)	0.0 (0)	**3.4 (12)**	0.0 (0)	**5.6 (19)**	0.0 (0)	**3.6 (11)**	0.0 (0)	0.0 (0)	0.0 (0)
Z4201	hypothetical protein		LB-antibiotics	1 (0)	2.8 (1)	0.0 (0)	0.0 (0)	0.0 (0)	**5.6 (3)**	0.0 (0)	0.0 (0)	0.0 (0)	3.0 (1)	0.0 (0)
Z5018	restricted to *Escherichia* and *Salmonella*		LB-antibiotics	1 (1)	1.5 (2)	3.2 (2)	0.4 (1)	1.5 (3)	**5.1 (11)**	−2.1 (0)	2.4 (4)	2.1 (3)	−2.1 (0)	−2.1 (0)
Z5071	inner membrane protein		LB-antibiotics	1 (4)	−0.7 (1)	1.9 (2)	0.5 (5)	−0.7 (2)	**5.7 (51)**	**2.4 (7)**	−4.4 (0)	1.2 (5)	0.5 (3)	−4.4 (0)
Z5212	hypothetical protein		LB-antibiotics	1 (0)	0.0 (0)	0.0 (0)	0.0 (0)	0.0 (0)	**5.6 (2)**	0.0 (0)	0.0 (0)	0.0 (0)	0.0 (0)	0.0 (0)
Z5214	*espY5′*, orphan, secreted protein	[39–41]	LB-antibiotics	1 (2)	−1.5 (0)	2.1 (1)	−0.6 (1)	−0.6 (1)	**6.0 (31)**	1.6 (2)	**−5.2 (0)**	−1.8 (0)	−1.2 (0)	**2.7 (2)**
Z5339	hypothetical protein		LB-antibiotics	1 (3)	1.3 (4)	2.4 (3)	1.4 (7)	1.7 (9)	**5.1 (26)**	−2.9 (0)	−2.9 (0)	1.9 (6)	1.1 (3)	**3.9 (8)**
Z2783	hypothetical protein		LB-solid	1 (1)	0.7 (1)	−2.1 (0)	0.4 (1)	0.6 (1)	−2.1 (0)	**5.2 (8)**	−2.1 (0)	**2.7 (2)**	**3.1 (3)**	−2.1 (0)
Z4570	hypothetical protein		LB-solid	1 (11)	**2.4 (34)**	0.6 (3)	**2.7 (61)**	1.4 (26)	−0.1 (3)	**5.6 (176)**	**4.2 (123)**	**4.3 (120)**	**2.1 (28)**	**4.7 (48)**
Z0359	hypothetical protein		minimal medium	1 (0)	−1.4 (0)	−1.4 (0)	−1.4 (0)	−1.4 (0)	−1.4 (0)	2.8 (1)	**5.4 (13)**	−1.4 (0)	1.6 (1)	−1.4 (0)
Z0360	hypothetical protein		minimal medium	1 (2)	0.1 (2)	−3.5 (0)	−3.5 (0)	1.6 (7)	2.1 (3)	−3.5 (0)	**5.2 (60)**	−3.5 (0)	**2.4 (8)**	2.3 (2)
Z0726	hypothetical protein		minimal medium	1 (3)	2.0 (8)	−2.6 (0)	2.2 (14)	**3.6 (41)**	−2.6 (0)	1.6 (3)	**7.5 (400)**	−2.6 (0)	−2.6 (0)	**4.8 (17)**
Z1519	hypothetical protein		minimal medium	1 (10)	**2.1 (26)**	**2.6 (10)**	0.8 (15)	1.6 (29)	0.6 (4)	−0.3 (3)	**5.0 (204)**	−0.8 (3)	**3.0 (45)**	−0.2 (1)
Z1521	hypothetical protein		minimal medium	1 (11)	**1.7 (24)**	**2.4 (10)**	0.2 (12)	1.6 (33)	−6.9 (0)	0.2 (5)	**5.6 (356)**	−0.4 (5)	**1.9 (25)**	−6.9 (0)
Z1966	hypothetical protein		minimal medium	1 (1)	1.0 (1)	−2.6 (0)	−2.6 (0)	2.3 (3)	−2.6 (0)	−2.6 (0)	**5.8 (26)**	−2.6 (0)	−2.6 (0)	3.2 (1)
Z2005	hypothetical protein		minimal medium	1 (0)	**3.7 (2)**	0.0 (0)	2.5 (1)	**3.6 (2)**	0.0 (0)	0.0 (0)	**5.5 (6)**	3.3 (1)	0.0 (0)	0.0 (0)
Z2511	hypothetical protein		minimal medium	1 (0)	0.0 (0)	0.0 (0)	0.0 (0)	**3.6 (4)**	0.0 (0)	0.0 (0)	**5.1 (7)**	0.0 (0)	3.0 (1)	0.0 (0)
Z3065	hypothetical protein		minimal medium	1 (19)	0.1 (13)	−6.7 (0)	−1.4 (6)	**4.0 (297)**	1.5 (15)	1.6 (21)	**8.9 (6063)**	**2.1 (49)**	0.6 (17)	**4.6 (80)**
Z3066	hypothetical protein		minimal medium	1 (1)	−1.4 (0)	−1.4 (0)	−1.4 (0)	**4.1 (37)**	−1.4 (0)	−1.4 (0)	**7.9 (337)**	−1.4 (0)	−1.4 (0)	−1.4 (0)
Z4912	hypothetical protein		minimal medium	1 (5)	1.5 (8)	2.9 (6)	0.9 (7)	**3.5 (51)**	−3.4 (0)	0.8 (3)	**7.4 (532)**	1.4 (7)	**2.6 (17)**	−3.4 (0)
Z4915	hypothetical protein		minimal medium	1 (2)	−0.5 (1)	−3.2 (0)	0.1 (2)	**2.3 (11)**	−3.2 (0)	1.0 (2)	**6.9 (181)**	1.0 (3)	1.7 (4)	−3.2 (0)
Z4917	hypothetical protein		minimal medium	1 (3)	1.7 (6)	1.3 (1)	−1.5 (1)	**2.9 (23)**	−4.0 (0)	2.2 (5)	**6.7 (207)**	−0.7 (1)	0.5 (3)	2.9 (4)
Z5122	hypothetical protein		minimal medium	1 (175)	−1.7 (35)	**−3.5 (3)**	**−5.4 (3)**	0.5 (245)	**3.9 (743)**	−0.3 (52)	**5.6 (5816)**	−1.3 (45)	**−4.8 (4)**	−2.0 (8)
Z2156	hypothetical protein		spinach	1 (17)	−1.1 (4)	−4.7 (0)	**−4.7 (0)**	−0.6 (10)	0.9 (8)	−4.7 (0)	**4.4 (221)**	**5.5 (448)**	1.2 (23)	−4.7 (0)
Z3271	hypothetical protein		spinach	1 (9)	−0.5 (4)	−4.6 (0)	−0.3 (6)	0.7 (14)	−4.6 (0)	**4.7 (79)**	**2.9 (41)**	**5.2 (191)**	0.8 (9)	**2.8 (10)**
Z3560	hypothetical protein		spinach	1 (1)	1.7 (2)	−3.4 (0)	−0.1 (1)	0.7 (1)	−3.4 (0)	**3.7 (4)**	**2.7 (4)**	**5.5 (25)**	1.1 (1)	−3.4 (0)
Z4375	hypothetical protein		spinach	1 (4)	1.1 (6)	−4.1 (0)	−1.6 (1)	1.4 (11)	−4.1 (0)	1.7 (5)	1.8 (9)	**5.0 (85)**	0.4 (3)	2.8 (5)
Z4376	hypothetical protein		spinach	1 (7)	0.2 (5)	1.0 (2)	0.7 (9)	**2.8 (44)**	1.3 (4)	1.4 (6)	**2.2 (18)**	**6.4 (320)**	**2.0 (15)**	1.5 (3)
Z4601	hypothetical protein		spinach	1 (0)	2.8 (2)	0.0 (0)	2.5 (3)	0.0 (0)	0.0 (0)	**4.2 (4)**	**4.6 (9)**	**6.6 (36)**	**3.9 (5)**	0.0 (0)
Z4890	hypothetical protein		spinach	1 (17)	**2.1 (45)**	−0.4 (2)	**3.9 (230)**	**4.9 (500)**	−0.7 (3)	−2.1 (1)	**4.7 (286)**	**5.6 (485)**	**2.1 (42)**	0.8 (5)
Z4909	hypothetical protein		spinach	1 (15)	0.7 (14)	−5.9 (0)	**2.7 (80)**	**2.5 (77)**	−5.9 (0)	**−5.9 (0)**	**2.6 (54)**	**5.2 (301)**	0.8 (14)	−5.9 (0)
Z5730	hypothetical protein		spinach	1 (4)	−3.3 (0)	−3.3 (0)	1.0 (7)	1.9 (14)	−3.3 (0)	−3.3 (0)	1.3 (6)	**5.1 (81)**	1.6 (7)	−3.3 (0)
Z0351	hypothetical protein		radish	1 (0)	**4.2 (1)**	0.0 (0)	0.0 (0)	**4.6 (3)**	0.0 (0)	0.0 (0)	0.0 (0)	3.3 (1)	**5.7 (4)**	0.0 (0)
Z1023	*ybiJ*, biofilm formation	[42, 43]	radish	1 (17)	−0.2 (8)	0.2 (3)	−2.6 (2)	1.5 (45)	**2.5 (25)**	−0.9 (3)	1.7 (35)	1.7 (31)	**6.6 (945)**	**3.3 (28)**
Z1027	*ybiM*, biofilm formation	[44]	radish	1 (17)	−0.3 (8)	−5.6 (0)	**−3.1 (1)**	−0.3 (12)	0.0 (4)	**−5.6 (0)**	**−5.6 (0)**	−2.3 (2)	**7.5 (1751)**	−5.6 (0)
Z1511	hypothetical protein		radish	1 (0)	0.0 (0)	0.0 (0)	0.0 (0)	0.0 (0)	0.0 (0)	0.0 (0)	0.0 (0)	0.0 (0)	**6.2 (5)**	0.0 (0)
Z4396	*ygiD*, biofilm formation	[45]	radish	1 (11)	0.8 (13)	−6.3 (0)	1.7 (33)	1.0 (24)	0.9 (6)	**−6.3 (0)**	1.3 (19)	1.0 (14)	**5.6 (337)**	1.5 (6)
Z4455	hypothetical protein		radish	1 (4)	1.0 (5)	−3.9 (0)	**4.2 (58)**	2.0 (14)	−3.9 (0)	−3.9 (0)	−3.9 (0)	**2.3 (11)**	**5.1 (79)**	3.0 (5)
Z4460	hypothetical protein		radish	1 (5)	−2.1 (1)	−4.8 (0)	−1.5 (1)	−0.3 (4)	1.8 (5)	−0.6 (1)	−4.8 (0)	0.9 (6)	**5.9 (197)**	**3.6 (10)**
Z4807	*yhhW*, quercetin detoxification	[46]	radish	1 (13)	0.0 (8)	0.7 (4)	**2.1 (47)**	0.4 (17)	1.0 (7)	**−6.2 (0)**	0.9 (16)	1.7 (25)	**5.8 (430)**	−0.3 (2)
Z5808	*yjfY*, biofilm formation	[47–49]	radish	1 (13)	**2.7 (50)**	1.5 (6)	0.4 (15)	**3.0 (99)**	1.8 (12)	**2.1 (19)**	−0.3 (7)	1.8 (27)	**7.5 (1365)**	**4.5 (49)**
Z0245	hypothetical protein		feces	1 (0)	**3.7 (2)**	0.0 (0)	**3.4 (2)**	0.0 (0)	0.0 (0)	0.0 (0)	0.0 (0)	3.3 (1)	3.0 (1)	**5.8 (3)**
Z0387	annotated as “dubious”		feces	1 (3)	0.1 (2)	**4.2 (12)**	−2.6 (0)	0.1 (3)	3.0 (8)	2.5 (7)	1.0 (4)	−2.6 (0)	−2.6 (0)	**6.0 (40)**
Z0706	hypothetical protein		feces	1 (0)	0.0 (0)	0.0 (0)	0.0 (0)	0.0 (0)	0.0 (0)	0.0 (0)	0.0 (0)	0.0 (0)	0.0 (0)	**5.8 (3)**
Z0742	methionine biosynthesis	[50–52]	feces	1 (4)	0.1 (2)	−5.1 (0)	0.5 (5)	0.9 (7)	−5.1 (0)	−5.1 (0)	**4.8 (68)**	1.7 (7)	1.6 (7)	**5.1 (23)**
Z1197	hypothetical protein		feces	1 (0)	0.0 (0)	0.0 (0)	2.5 (3)	0.0 (0)	0.0 (0)	0.0 (0)	**4.6 (9)**	3.3 (3)	0.0 (0)	**5.8 (6)**
Z1517	hypothetical protein		feces	1 (0)	0.0 (0)	0.0 (0)	**3.4 (7)**	0.0 (0)	0.0 (0)	0.0 (0)	0.0 (0)	0.0 (0)	0.0 (0)	**5.8 (8)**
Z1527	switches between biofilm and planctonic life style, *ycdT*	[53–55]	feces	1 (7)	−0.6 (3)	−6.4 (0)	−0.7 (4)	0.5 (10)	1.2 (5)	0.8 (4)	**2.5 (28)**	0.9 (8)	−1.0 (2)	**5.4 (56)**
Z2119	hypothetical protein		feces	1 (0)	0.0 (0)	0.0 (0)	2.5 (3)	0.0 (0)	0.0 (0)	0.0 (0)	0.0 (0)	0.0 (0)	0.0 (0)	**5.8 (8)**
Z2199	hypothetical protein		feces	1 (0)	0.0 (0)	0.0 (0)	0.0 (0)	2.7 (3)	0.0 (0)	0.0 (0)	0.0 (0)	0.0 (0)	0.0 (0)	**5.8 (6)**
Z2368	encoded within prophage CP-933R		feces	1 (5)	0.2 (3)	−2.6 (0)	0.8 (7)	1.0 (10)	−2.6 (0)	**4.4 (37)**	−2.6 (0)	−2.6 (0)	1.9 (11)	**6.8 (104)**
Z2560	hypothetical protein		feces	1 (6)	−0.4 (3)	0.7 (2)	0.2 (6)	−0.1 (5)	1.0 (3)	1.9 (7)	−1.0 (2)	**2.0 (14)**	**−4.6 (0)**	**5.0 (33)**
Z2619	membrane protein, glucuronate metabol.	[38, 56]	feces	1 (0)	−1.4 (0)	−1.4 (0)	**3.2 (2)**	2.2 (1)	−1.4 (0)	−1.4 (0)	3.1 (2)	−1.4 (0)	2.5 (1)	**6.0 (4)**
Z3722	contains functional domain		feces	1 (21)	0.7 (21)	1.8 (12)	−1.0 (9)	0.8 (35)	**−7.4 (0)**	1.0 (14)	1.3 (33)	−1.4 (5)	**2.5 (70)**	**5.2 (129)**

^1^For each gene, the first number indicates the logFC of a certain condition compared to LB; RPKM values are shown in parentheses. Significantly differentially expressed genes are in bold (i.e., p values ≤ 0.05 in *edgeR*). For a graphic version, indicating the magnitude of the absolute value of logFC in shades of grey, see Additional file 4.

### Transcription of virulence genes

The LEE (Locus of Enterocyte Effacement) pathogenicity island comprises 41 genes responsible for the attachment of EHECs to mammalian host cells and effacing lesions [57, 58]. Table 3 (for a graphic version see Additional file 4) summarizes their regulation. The most prominent up-regulated LEE gene is the secreted effector protein gene *espZ* (Z5122) in minimal medium (logFC > 5 compared to LB medium). It interacts with several host proteins (see [59–61]). The extremely high transcription level of *espZ* in minimal medium is quite surprising since it is the only medium completely lacking host cell related compounds. Most other LEE genes encoding the type III secretion system (TTSS) (e.g. Z5132 – Z5135), some translocated proteins like EspG (Z5142), EspH (Z5115), intimin (*eae*, Z5110), transcriptional regulators (e.g. *ler*, Z5140), and the chaperone CesD (Z5127) also display high transcript levels (RPKMs) in minimal medium and are also active in LB-antibiotics (Table 3).

**Table 3:** **Genes of the LEE pathogenicity island** ^**1**^

Gene tag	Product	LB	LB-pH9	LB-pH4	LB-15°C	LB-nitrite	LB-antibiotics	LB-solid	minimal medium	spinach juice	radish sprouts	feces
Z5100	hypothetical protein	1 (20)	**−6.8 (0)**	−1.5 (1)	**−6.8 (0)**	**−3.2 (2)**	1.8 (17)	**−6.8 (0)**	−1.0 (6)	**−3.5 (1)**	**−6.8 (0)**	−6.8 (0)
Z5102	hypothetical protein	1 (149)	**−5.5 (2)**	−0.7 (15)	**−4.9 (4)**	**−4.1 (7)**	0.1 (41)	**−8.3 (0)**	−0.2 (81)	**−4.1 (5)**	**−3.4 (8)**	**−8.3 (0)**
Z5103	hypothetical protein	1 (128)	**−3.0 (10)**	**−7.9 (0)**	**−4.0 (7)**	**−3.0 (15)**	1.7 (117)	**−7.9 (0)**	1.1 (187)	**−3.6 (6)**	**−4.9 (2)**	**−7.9 (0)**
Z5104	hypothetical protein	1 (463)	**−3.9 (17)**	**−3.6 (6)**	**−2.6 (63)**	**−2.2 (91)**	−0.5 (80)	**−3.7 (11)**	0.6 (431)	**−4.3 (13)**	**−3.3 (26)**	**−4.6 (3)**
Z5105	secreted protein EspB	1 (903)	**−3.5 (46)**	**−4.6 (6)**	**−4.5 (35)**	−1.5 (302)	**−2.0 (57)**	**−3.8 (21)**	0.9 (1070)	**−4.3 (26)**	**−3.1 (55)**	**−6.8 (1)**
Z5106	secreted protein EspD	1 (847)	**−4.8 (19)**	**−5.0 (4)**	**−6.2 (10)**	**−2.8 (119)**	−0.5 (155)	**−4.3 (15)**	1.0 (1070)	**−4.4 (24)**	**−3.8 (32)**	**−12.9 (0)**
Z5107	secreted protein EspA	1 (1035)	**−4.5 (31)**	**−7.0 (1)**	**−7.7 (4)**	**−2.7 (161)**	1.6 (888)	**−3.1 (45)**	1.4 (1819)	**−4.1 (37)**	**−3.4 (58)**	**−12.3 (0)**
Z5108	hypothetical protein	1 (324)	**−4.8 (7)**	**−5.1 (2)**	**−11.4 (0)**	**−3.2 (34)**	0.2 (97)	**−3.4 (11)**	0.8 (347)	**−4.5 (8)**	**−4.0 (12)**	**−3.0 (7)**
Z5109	hypothetical protein	1 (32)	**−2.6 (3)**	**−8.2 (0)**	**−5.7 (0)**	**−2.9 (4)**	−1.0 (4)	−0.6 (7)	1.4 (52)	**−3.0 (2)**	**−3.3 (2)**	−2.3 (1)
Z5110	intimin adherence protein	1 (392)	**−4.7 (9)**	**−4.5 (3)**	**−5.7 (6)**	**−3.1 (45)**	1.2 (244)	**−3.9 (9)**	0.6 (376)	**−4.6 (9)**	**−3.4 (20)**	**−7.3 (0)**
Z5111	hypothetical protein	1 (363)	**−5.8 (4)**	**−5.1 (2)**	**−5.6 (7)**	**−3.5 (31)**	**2.6 (608)**	**−4.7 (5)**	1.1 (523)	**−4.9 (7)**	**−3.9 (13)**	**−10.4 (0)**
Z5112	putative translocated intimin receptor protein	1 (247)	**−3.4 (15)**	**−4.4 (2)**	**−4.8 (8)**	−1.8 (67)	1.2 (149)	**−2.9 (11)**	**1.9 (594)**	**−3.5 (12)**	**−3.2 (15)**	**−11.7 (0)**
Z5113	hypothetical protein	1 (337)	**−3.0 (25)**	−2.0 (15)	**−2.9 (40)**	−1.7 (103)	1.1 (194)	**−2.3 (23)**	**1.7 (689)**	**−2.5 (36)**	**−4.2 (10)**	**−3.3 (6)**
Z5114	hypothetical protein	1 (55)	**−2.3 (7)**	−0.2 (9)	**−2.9 (7)**	−1.8 (16)	**2.7 (102)**	−1.3 (8)	**2.3 (183)**	−2.0 (9)	**−3.5 (3)**	**−7.5 (0)**
Z5115	hypothetical protein	1 (111)	**−2.9 (10)**	−2.0 (5)	**−3.6 (8)**	**−1.9 (30)**	1.5 (90)	−1.3 (16)	**1.6 (231)**	**−2.7 (11)**	**−2.8 (9)**	−0.1 (19)
Z5116	hypothetical protein	1 (60)	**−2.6 (6)**	**−2.6 (2)**	**−5.5 (1)**	**−2.9 (8)**	1.4 (44)	−0.4 (16)	0.8 (71)	**−4.1 (2)**	**−2.1 (9)**	0.3 (13)
Z5117	hypothetical protein	1 (53)	**−4.1 (2)**	**−6.9 (0)**	**−4.4 (2)**	**−2.7 (7)**	1.1 (29)	−2.7 (3)	0.6 (52)	**−2.6 (5)**	**−6.9 (0)**	−6.9 (0)
Z5118	hypothetical protein	1 (69)	**−4.9 (1)**	−2.3 (2)	**−7.6 (0)**	**−4.0 (4)**	1.1 (39)	**−7.6 (0)**	−0.1 (38)	**−2.8 (5)**	**−2.4 (7)**	**−7.6 (0)**
Z5119	hypothetical protein	1 (79)	**−2.2 (10)**	**−3.4 (1)**	**−3.4 (6)**	**−2.0 (19)**	0.5 (29)	**−2.2 (6)**	0.8 (84)	**−3.7 (4)**	**−3.7 (4)**	−0.9 (7)
Z5120	hypothetical protein	1 (37)	**−3.1 (3)**	**−3.0 (1)**	**−3.5 (3)**	**−2.3 (7)**	0.7 (17)	−1.5 (4)	**1.8 (83)**	**−2.7 (3)**	**−3.8 (2)**	**−9.3 (0)**
Z5121	hypothetical protein	1 (29)	**−3.8 (1)**	−6.5 (0)	**−4.0 (1)**	−2.0 (8)	**2.1 (37)**	−1.3 (4)	**1.9 (79)**	−1.3 (8)	**−6.5 (0)**	0.9 (10)
Z5122	hypothetical protein	1 (175)	−1.7 (35)	**−3.5 (3)**	**−5.4 (3)**	0.5 (245)	**3.9 (743)**	−0.3 (52)	**5.6 (5816)**	−1.3 (45)	**−4.8 (4)**	−2.0 (8)
Z5123	hypothetical protein	1 (41)	**−3.4 (2)**	−1.8 (2)	**−7.1 (0)**	−1.7 (11)	**2.8 (72)**	−1.3 (5)	**2.7 (166)**	−1.9 (6)	**−7.1 (0)**	−7.1 (0)
Z5124	hypothetical protein	1 (37)	**−3.8 (2)**	**−7.4 (0)**	**−4.9 (1)**	−1.4 (13)	**3.0 (80)**	−2.2 (3)	**2.2 (106)**	−1.2 (9)	**−2.5 (4)**	**−7.4 (0)**
Z5125	hypothetical protein	1 (80)	**−4.0 (3)**	**−8.2 (0)**	**−4.3 (3)**	−1.4 (30)	**1.8 (75)**	**−4.0 (2)**	**1.8 (186)**	−2.0 (12)	**−4.3 (2)**	−2.4 (3)
Z5126	hypothetical protein	1 (50)	**−4.4 (1)**	**−4.0 (1)**	**−6.8 (0)**	**−2.4 (9)**	**2.5 (78)**	−1.2 (8)	**1.7 (104)**	**−2.8 (4)**	**−4.1 (2)**	**−9.3 (0)**
Z5127	hypothetical protein	1 (91)	**−4.3 (3)**	**−8.5 (0)**	**−8.5 (0)**	**−2.6 (15)**	1.7 (82)	**−2.7 (5)**	1.2 (143)	**−2.5 (10)**	**−3.6 (5)**	**−8.5 (0)**
Z5128	hypothetical protein	1 (74)	**−2.5 (7)**	**−7.9 (0)**	**−5.4 (1)**	**−5.2 (2)**	**3.1 (165)**	−2.2 (5)	−0.1 (44)	−1.6 (15)	**−2.7 (6)**	**−7.9 (0)**
Z5129	negative regulator GrlR	1 (118)	**−2.4 (14)**	**−8.5 (0)**	**−5.1 (3)**	**−3.9 (7)**	**3.7 (429)**	−0.5 (30)	0.9 (148)	−1.0 (36)	**−4.6 (3)**	−2.7 (3)
Z5131	hypothetical protein	1 (12)	**−5.5 (0)**	−5.5 (0)	−2.1 (2)	**−2.8 (1)**	**3.4 (35)**	−0.3 (3)	1.0 (16)	−1.2 (3)	**−5.5 (0)**	1.3 (5)
Z5132	secretion system apparatus protein SsaU	1 (41)	**−5.5 (0)**	**−8.3 (0)**	**−8.3 (0)**	**−4.7 (1)**	0.1 (11)	**−8.3 (0)**	**−2.5 (4)**	**−5.0 (1)**	**−4.3 (1)**	**−8.3 (0)**
Z5133	hypothetical protein	1 (47)	**−4.5 (1)**	**−8.2 (0)**	**−5.7 (1)**	**−5.5 (1)**	0.2 (15)	−1.7 (5)	−1.9 (8)	**−2.7 (4)**	**−5.2 (1)**	**−8.2 (0)**
Z5134	hypothetical protein	1 (62)	**−7.1 (0)**	**−7.1 (0)**	**−7.1 (0)**	**−4.4 (3)**	−1.5 (6)	**−2.9 (3)**	−1.6 (13)	**−3.8 (2)**	**−7.1 (0)**	−7.1 (0)
Z5135	type III secretion system protein	1 (95)	**−6.2 (1)**	**−8.9 (0)**	**−8.9 (0)**	**−3.6 (7)**	0.6 (37)	**−2.2 (7)**	−1.0 (29)	**−4.7 (2)**	**−5.0 (2)**	**−8.9 (0)**
Z5136	hypothetical protein	1 (149)	**−9.5 (0)**	**−9.5 (0)**	**−6.1 (2)**	**−4.3 (7)**	−0.3 (32)	**−4.3 (2)**	−0.7 (56)	**−5.2 (2)**	**−4.0 (5)**	**−9.5 (0)**
Z5137	hypothetical protein	1 (232)	**−5.2 (4)**	**−3.2 (4)**	**−4.8 (7)**	**−4.0 (14)**	0.5 (84)	**−2.8 (11)**	−0.8 (84)	**−4.1 (8)**	**−3.1 (15)**	**−4.3 (2)**
Z5138	hypothetical protein	1 (438)	**−5.4 (5)**	**−3.7 (5)**	**−10.0 (0)**	**−4.5 (17)**	−0.8 (60)	**−3.5 (12)**	−1.6 (84)	**−5.2 (6)**	**−4.1 (13)**	**−3.2 (8)**
Z5139	hypothetical protein	1 (899)	**−6.8 (4)**	**−10.4 (0)**	**−10.4 (0)**	**−5.6 (16)**	−1.2 (89)	**−2.8 (37)**	**−2.6 (82)**	**−5.6 (9)**	**−5.2 (12)**	**−3.6 (11)**
Z5140	hypothetical protein	1 (597)	**−4.0 (23)**	**−3.2 (11)**	**−10.9 (0)**	**−3.1 (67)**	0.6 (246)	−1.8 (59)	−0.9 (208)	**−4.3 (17)**	**−4.0 (20)**	**−3.4 (9)**
Z5142	hypothetical protein	1 (51)	−1.8 (9)	−2.0 (2)	**−2.8 (6)**	−1.0 (23)	−0.1 (12)	−1.6 (6)	1.2 (75)	−**2.5 (5)**	**−2.3 (6)**	−1.5 (3)
Z5143	hypothetical protein	1 (6)	**−5.2 (0)**	−5.2 (0)	**−5.2 (0)**	**−5.2 (0)**	−5.2 (0)	−5.2 (0)	1.6 (11)	**−5.2 (0)**	**−5.2 (0)**	−5.2 (0)

^1^For each gene, the first number indicates the logFC of a certain condition compared to LB; RPKM values are shown in parentheses. Significantly differentially expressed genes are in bold (i.e., p values ≤ 0.05 in *edgeR*). For a graphic version, indicating the magnitude of the absolute value of logFC in shades of grey, see Additional file 4.

Furthermore, 62 non-LEE encoded, virulence associated genes [39, 62] were found to be up-regulated in the absence of a host (Additional file 5: Table S5). Several of them locate to prophages and are secreted effector proteins. Similar to the LEE encoded genes, expression levels of most of these 62 additional genes are highest in LB medium. The remaining, especially in feces, have logFCs between 1 and 8 under other conditions compared to LB. We assume that LB’s ingredients, a tryptic digest of casein and yeast extract from autolysates, mimics vertebrate host-like conditions. Stress, like alkaline pH and nitrite, completely represses the induction of all LEE genes and many other virulence genes. Furthermore, these virulence-associated genes appear not to be active on radish sprouts as well as in spinach medium at the time point of harvest. Though EHECs are known to proliferate on plant surfaces [63], the TTSS seems to play no role in a prolonged EHEC-plant interaction.

### Gene expression in the presence of antibiotics

LB-antibiotics is the condition with the lowest number of active genes. Among the highly up-regulated genes (logFC ≥ 5), 70% originate from prophages CP-933H, CP-933K, BP933W, CP-933C, CP-933X, CP-933U, and CP-933V. Interestingly, LB-antibiotics is the condition with the highest number of hypothetical genes being induced. Sixteen of the 26 highly antibiotic-induced hypotheticals are encoded by prophages. Z0314 and Z0316 are from prophage CP-933H and have high similarities to phage tail fiber proteins. The other 14 genes originate from different prophages and their function is unknown. However, they were also active after treatment with norfloxacin [37]. Z1434 was also identified after a human infection using the *in vivo*-induced antigen technology (IVIAT; [38]). For the other hypotheticals, no experimental data exist. By a bioinformatic approach, Z5214 was identified as a secreted effector protein, espY5’ [39]. While most prophages of *Escherichia coli* O157:H7 are regarded to be defective, Asadulghani *et al.*[64] reported that these phages are still inducible. Antibiotics activate the SOS-response, thereby inducing phage replication. Therefore, it is not surprising that a higher number of phage-borne hypothetical genes are active.

It is known that the treatment of an EHEC infection with antibiotics may potentiate the severity of the disease. Among clinically applied antibiotics, the combination of trimethoprim with sulfamethoxazole seems to be the worst choice [3]. Interestingly, this antibiotic mixture strongly induces transcription of CP-933V and BP-933W. These two phages encode the shiga-toxins which contribute essentially to the clinical symptoms of an infection [65]. Their activation provides a direct explanation for the high rate in clinical complications. Furthermore, the LEE pathogenicity island is also active in LB-antibiotics (see Table 3). In some studies, a connection of the regulation of phages and the LEE pathogenicity island was found (e.g., [66, 67]). Phage-encoded regulators have effects on the activity of TTSS and LEE genes respectively.

### Transcription of genes in cattle feces

#### Annotated genes active in cattle feces

The gastrointestinal tract of ruminants is considered a major reservoir of *Escherichia coli* O157:H7 [68]. However, no transcriptomes under this condition have been reported. We could detect several genes up regulated in feces compared to LB (Table 4, for a graphic version see Additional file 4). A highly up-regulated gene in cattle feces is *glgS* with a logFC of 6.6. It is a central gene in glycogen metabolism: this metabolite accumulates under starvation [69]. Other highly active metabolic enzymes are *idi* (Z4227, isopentenyl-diphosphate delta-isomerase), a key enzyme of isoprenoid pathways, and *caiA* (Z0045, crotonobetainyl-CoA dehydrogenase). The latter is involved in the metabolism of L-carnitine, a ubiquitous compound in eukaryotic tissues, which is metabolized to γ-butyrobetaine in *E. coli*[70].

**Table 4:** **Genes compared to LB with high logFCs in feces** ^**1**^

Gene tag	Product	LB	LB-pH9	LB-pH4	LB-15°C	LB-nitrite	LB-antibiotics	LB-solid	minimal medium	spinach juice	radish sprouts	feces
Z0014	molecular chaperone DnaK	1 (185)	−1.0 (55)	0.1 (33)	1.6 (470)	1.6 (520)	0.5 (70)	0.8 (109)	0.0 (120)	**3.6 (1290)**	1.2 (248)	**3.7 (398)**
Z0015	chaperone protein DnaJ	1 (74)	−1.4 (16)	−1.4 (4)	1.3 (150)	0.8 (120)	−0.9 (10)	0.0 (23)	−0.5 (31)	0.9 (78)	1.2 (99)	**4.2 (220)**
Z0045	crotonobetainyl-CoA dehydrogenase	1 (7)	1.3 (10)	0.4 (1)	−0.1 (5)	0.9 (12)	0.7 (3)	**2.7 (14)**	1.2 (9)	**2.5 (23)**	−1.5 (1)	**5.8 (63)**
Z2477	thiosulfate:cyanide sulfurtransferase	1 (558)	0.4 (470)	−0.1 (94)	0.9 (963)	0.1 (603)	**−2.3 (31)**	−0.7 (121)	**−2.2 (83)**	−1.6 (113)	−0.4 (252)	**2.4 (537)**
Z2478	peripheral inner membrane phage-shock protein	1 (17)	1.4 (28)	−4.9 (0)	**2.0 (60)**	1.2 (37)	−4.9 (0)	1.6 (17)	0.6 (16)	0.6 (15)	**4.3 (198)**	**7.7 (598)**
Z2479	DNA-binding transcriptional activator PspC	1 (17)	1.5 (31)	−0.4 (2)	**1.9 (57)**	1.4 (46)	−0.1 (5)	**−5.7 (0)**	0.8 (20)	−0.9 (6)	**4.1 (182)**	**7.2 (470)**
Z2480	phage shock protein B	1 (25)	1.4 (39)	−0.2 (4)	**1.9 (80)**	1.2 (55)	−5.5 (0)	−0.3 (7)	−0.9 (8)	0.0 (15)	**3.9 (228)**	**6.8 (482)**
Z2482	phage shock protein PspA	1 (64)	0.8 (63)	0.8 (18)	**2.0 (206)**	0.5 (81)	−0.4 (12)	0.6 (31)	0.6 (59)	0.6 (56)	**4.8 (1077)**	**6.5 (970)**
Z2611	DNA replication terminus site-binding protein Tus	1 (9)	−0.4 (4)	1.3 (4)	**1.8 (26)**	0.8 (15)	−6.0 (0)	0.2 (3)	−1.4 (2)	−1.2 (2)	0.7 (9)	**5.0 (47)**
Z2876	heat shock protein HtpX	1 (93)	1.4 (154)	0.2 (19)	**1.8 (284)**	1.0 (187)	−0.2 (22)	**3.0 (262)**	0.0 (60)	−0.3 (46)	0.1 (59)	**3.9 (246)**
Z2900	DNA damage-inducible protein YebG	1 (112)	−0.1 (61)	1.3 (42)	−0.1 (83)	0.3 (123)	**2.8 (196)**	0.8 (59)	0.6 (99)	0.0 (60)	0.8 (113)	**3.7 (226)**
Z3886	protein disaggregation chaperone ClpB	1 (239)	−1.6 (44)	**2.4 (194)**	0.5 (267)	0.3 (265)	−0.1 (56)	0.9 (140)	−0.4 (106)	**2.7 (827)**	1.1 (292)	**2.5 (216)**
Z4227	isopentenyl-diphosphate delta-isomerase	1 (22)	**1.9 (52)**	0.3 (5)	1.2 (43)	**2.0 (89)**	−6.6 (0)	1.2 (18)	0.2 (16)	1.6 (40)	**1.5 (38)**	**5.6 (180)**
Z4291	16S ribosomal RNA methyltransferase RsmE	1 (57)	−1.0 (17)	1.0 (18)	−0.9 (26)	−0.8 (30)	0.9 (27)	−1.4 (7)	−0.1 (33)	1.1 (70)	0.0 (34)	**4.1 (165)**
Z4401	glycogen synthesis protein GlgS	1 (240)	0.4 (182)	−0.2 (32)	−0.4 (141)	1.2 (480)	−0.3 (47)	**4.0 (1226)**	0.5 (196)	−0.3 (104)	**2.6 (838)**	**6.6 (3617)**
Z5182	heat shock chaperone IbpB	1 (26)	−0.2 (13)	−0.1 (4)	0.4 (29)	0.3 (29)	1.5 (19)	**2.5 (47)**	−0.3 (13)	1.0 (30)	**2.4 (80)**	**5.7 (224)**
Z5183	heat shock protein IbpA	1 (26)	0.5 (22)	1.7 (14)	**2.1 (96)**	1.2 (55)	1.5 (20)	**2.6 (54)**	0.1 (17)	**3.0 (125)**	**2.2 (72)**	**6.9 (509)**
Z5458	periplasmic repressor CpxP	1 (25)	**3.2 (127)**	1.7 (13)	0.0 (20)	1.1 (47)	**2.9 (45)**	**4.6 (191)**	−0.2 (13)	−2.3 (3)	**2.5 (84)**	**6.0 (260)**

^**1**^For each gene, the first number indicates the logFC of a certain condition compared to LB; RPKM values are shown in parentheses. Significantly differentially expressed genes are in bold (i.e., p values ≤ 0.05 in *edgeR*). For a graphic version, indicating the magnitude of the absolute value of logFC in shades of grey, see Additional file 4.

Many up-regulated genes are either involved in macromolecule-protection or associated to membrane stress. One example is the up-regulated phage shock regulon *pspEDCBA* (Z2477 – Z2482, logFCs between 2 and 8) which is known to respond to certain stress conditions such as phage attack, heat shock, hyperosmotic stress, or exposure to hydrophobic organic solvents [71]. Further, the co-chaperones *dnaK* (Z0014, Table 4) and *dnaJ* (Z0015) are active in feces with logFCs of 3.7 and 4.2, respectively. These chaperones are essential for the folding of newly synthesized proteins or refolding of misfolded proteins [72, 73]. A similar function in disaggregation and reactivation of proteins has the chaperone ClpB (additionally active in LB-pH4 and spinach juice [74]). A high logFC of these chaperone genes should indicate cellular stress. Other active stress related genes include *tus*, encoding a DNA replication termination protein [75], furthermore, *yebG*, which is involved in DNA-damage repair, and in addition the *ibpAB* operon, which plays a role in the recognition of aggregated proteins [76].

Membrane stress is indicated by CpxP (Z5458, formerly YiiO), a small protein located in the periplasm. The protein interacts with the *cpx*-regulon, a two component signal transduction system responsible for sensing envelope stress [77]. HtpX, a member of the σ^32^ heat-shock regulon, is involved in the degradation and dislocation of unassembled membrane proteins [78]. The highest up-regulation of this gene in feces indicates the presence of membrane stress. Interestingly, many of the up-regulated hypothetical genes in cattle feces also contain membrane domains.

#### Hypothetical genes active in cattle feces

Thirteen hypotheticals are only induced in cattle feces with a logFC higher than 5 (see Table 2). Z0387 and Z3722 are unknown genes which have never been reported to be active under any condition before. As in radish sprouts, several up-regulated genes are involved in biofilm formation, e.g. *ycdT*. Interestingly, the hypothetical gene Z2619 is similar to membrane proteins, probably involved in the uptake of host derived compounds. Z2619 has high similarities to UidC of *Escherichia coli* E101, belonging to the *uidRABC* operon which is involved in the metabolism of glucuronate, a molecule present in the gut [56]. Furthermore, there is experimental evidence based on *in vivo*-induced antigen technology (IVIAT) for *Escherichia coli* O157:H7, that Z2619 is also active during human infection [38].

In summary, most of the highly active genes in cattle feces are connected to membrane stress or involved in the protection or reactivation of proteins. Based on these findings we suggest that EHEC may be under considerable environmental stress in the colon of ruminants.

### Gene expression on radish sprouts

#### Utilization of carbon sources

**Figure 5:**
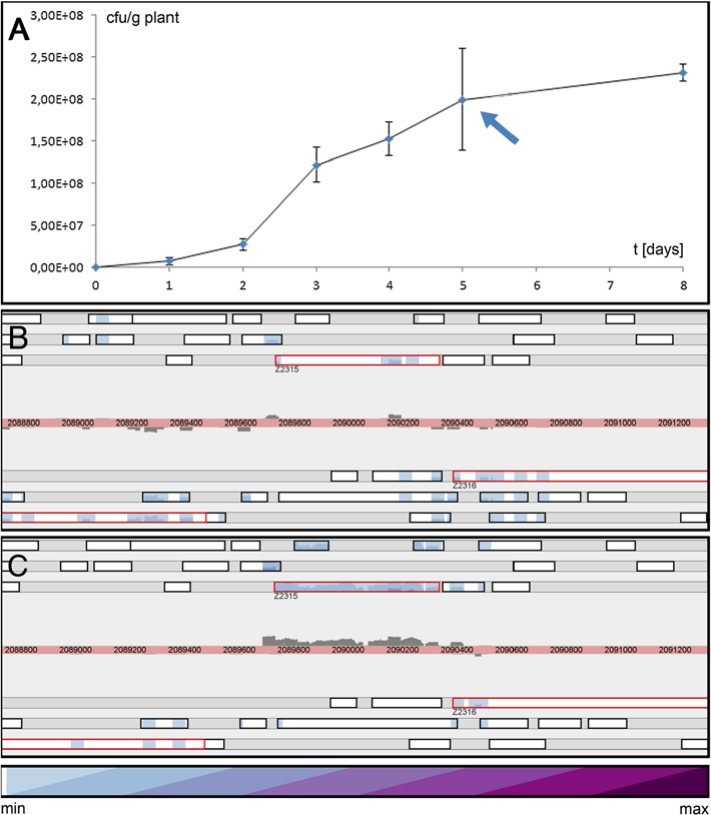
**Growth curve of EHEC on radish sprouts and expression of** ***azoR*** **. A:** growth on radish sprouts within 8 days. The sprouts were inoculated with 4 × 10^2^ cfu/g plant and harvested after 5 days during late exponential/early stationary phase (marked with arrow). **B:** expression of *azoR* (Z2315) in LB-pH9 (RPKM = 6, color map maximum value of 1 × 10^6^); for legend, see Figure 6. **C:** expression of azoR in radish sprouts (shown is the SOLiD replicate), *azoR* is highly covered with reads (RPKM = 190, color map maximum value of 3 × 10^5^); for legend, see Figure 6.

After growth on radish sprouts (Figure 5A), 997 genes have significantly different transcript levels (478 up/519 down) compared to LB medium. A distinctive pattern of genes with high transcription levels includes genes active in the degradation of fructose *fruAKB* (Z3425-Z3427; logFCs between 5 and 8), trehalose *otsAB* (Z2949, Z2950; logFCs between 2 and 4), and arabinose *araAHGF* (Z0070, Z2951, Z2953, Z2954), including Z3511-Z3513/Z3515 (Table 5, for a graphic version see Additional file 4). EHECs are able to utilize these plant-specific carbon sources. Plants are known to exudate certain carbon sources and other substances from their roots to maintain a certain microbiome, which in turn provides the plants with micronutrients [79].

**Table 5:** **Genes compared to LB either with highest logFC or RPKM values on radish sprouts and in spinach medium or genes known from an association to plants** ^**1**^

Gene tag	Product	LB	LB-pH9	LB-pH4	LB-15°C	LB-nitrite	LB-antibiotics	LB-solid	minimal medium	spinach juice	radish sprouts	feces
Z0070	L-arabinose isomerase	1 (4)	−0.5 (2)	1.2 (2)	−1.3 (1)	1.7 (13)	0.0 (1)	**−5.6 (0)**	−1.1 (1)	0.1 (3)	**3.1 (23)**	−5.6 (0)
Z1106	HCP oxidoreductase, NADH-dependent	1 (3)	−1.1 (1)	−4.8 (0)	**−4.8 (0)**	0.3 (4)	−4.8 (0)	0.4 (2)	0.7 (4)	**7.7 (443)**	**2.1 (9)**	1.1 (1)
Z1107	hydroxylamine reductase	1 (2)	0.2 (1)	−4.7 (0)	**2.1 (7)**	0.2 (2)	−4.7 (0)	1.5 (2)	−1.1 (1)	**8.3 (356)**	**2.3 (5)**	−4.7 (0)
Z1109	aquaporin Z	1 (9)	**3.0 (41)**	1.3 (4)	**4.1 (130)**	**3.4 (88)**	−5.6 (0)	−1.4 (1)	1.1 (12)	0.2 (6)	**3.8 (70)**	−5.6 (0)
Z1390	hydrogenase 1 large subunit	1 (2)	0.2 (1)	−5.0 (0)	−1.1 (1)	1.0 (4)	−5.0 (0)	−5.0 (0)	1.3 (4)	**7.6 (255)**	0.5 (2)	−5.0 (0)
Z1670	curli production assembly/transport component, 2nd curli operon	1 (24)	1.6 (43)	1.0 (8)	**2.1 (88)**	1.2 (54)	**−7.3 (0)**	**−3.1 (1)**	0.3 (18)	−0.9 (7)	1.2 (32)	0.5 (6)
Z1671	curli assembly protein CsgF	1 (7)	**2.3 (21)**	−4.6 (0)	**2.0 (26)**	−0.4 (5)	−4.6 (0)	−4.6 (0)	0.9 (9)	−4.6 (0)	**2.4 (24)**	−4.6 (0)
Z1672	curli assembly protein CsgE	1 (12)	**2.2 (35)**	**2.3 (11)**	1.5 (30)	0.9 (23)	−5.3 (0)	−0.1 (4)	1.2 (19)	−2.0 (2)	**2.2 (34)**	−5.3 (0)
Z1673	DNA-binding transcriptional regulator CsgD	1 (39)	**1.9 (83)**	1.6 (19)	0.0 (33)	0.4 (50)	**−7.6 (0)**	**−3.4 (1)**	0.6 (37)	**−3.4 (2)**	1.3 (56)	−1.8 (2)
Z1675	curlin minor subunit	1 (20)	**3.1 (114)**	−6.3 (0)	**2.9 (138)**	**2.9 (156)**	0.4 (7)	−1.1 (3)	**2.5 (75)**	1.2 (28)	**6.0 (803)**	−0.4 (3)
Z1676	cryptic curlin major subunit	1 (46)	**5.3 (1143)**	0.6 (13)	**2.0 (168)**	**3.3 (472)**	0.5 (18)	−0.5 (11)	**3.8 (440)**	−0.3 (23)	**5.6 (1336)**	**−7.5 (0)**
Z1697	biofilm formation regulatory protein BssS	1 (113)	**2.4 (386)**	**2.5 (116)**	**6.7 (10874)**	**4.4 (2326)**	1.5 (89)	**3.7 (512)**	1.5 (219)	**3.0 (556)**	**6.1 (4555)**	**4.7 (530)**
Z2243	nitrite extrusion protein 2	1 (0)	2.5 (1)	3.2 (1)	**5.8 (19)**	**4.4 (8)**	−2.1 (0)	−2.1 (0)	2.4 (1)	2.1 (1)	**7.0 (30)**	3.7 (1)
Z2315	azoreductase	1 (21)	−1.1 (6)	−0.4 (3)	0.5 (25)	−0.8 (11)	1.8 (19)	0.8 (12)	1.5 (39)	1.1 (25)	**4.1 (213)**	**2.9 (26)**
Z2479	DNA-binding transcriptional activator PspC	1 (17)	1.5 (31)	−0.4 (2)	**1.9 (57)**	1.4 (46)	−0.1 (5)	**−5.7 (0)**	0.8 (20)	−0.9 (6)	**4.1 (182)**	**7.2 (470)**
Z2480	phage shock protein B	1 (25)	1.4 (39)	−0.2 (4)	**1.9 (80)**	1.2 (55)	−5.5 (0)	−0.3 (7)	−0.9 (8)	0.0 (15)	**3.9 (228)**	**6.8 (482)**
Z2482	phage shock protein PspA	1 (64)	0.8 (63)	0.8 (18)	**2.0 (206)**	0.5 (81)	−0.4 (12)	0.6 (31)	0.6 (59)	0.6 (56)	**4.8 (1077)**	**6.5 (970)**
Z2591	acid shock protein precursor	1 (37)	−0.9 (13)	**5.3 (261)**	**−6.6 (0)**	−0.9 (20)	−6.6 (0)	**−6.6 (0)**	−1.5 (9)	**1.8 (80)**	**7.5 (4178)**	−6.6 (0)
Z2949	trehalose-6-phosphate synthase	1 (20)	**2.0 (49)**	0.3 (4)	0.9 (32)	**3.0 (151)**	−2.2 (1)	0.1 (7)	1.0 (26)	**2.3 (57)**	**2.6 (74)**	1.3 (9)
Z2950	trehalose-6-phosphate phosphatase	1 (16)	**2.7 (66)**	0.6 (4)	1.5 (41)	**3.2 (141)**	−1.1 (2)	−0.2 (5)	1.0 (20)	**2.2 (45)**	**3.7 (133)**	1.4 (8)
Z2951	partial high-affinity L-arabinose transport system; membrane protein, fragment 2	1 (4)	0.0 (3)	−4.6 (0)	−0.6 (2)	0.6 (6)	−4.6 (0)	−4.6 (0)	−4.6 (0)	−4.6 (0)	**2.3 (12)**	−4.6 (0)
Z2953	L-arabinose transporter ATP-binding protein	1 (8)	1.4 (13)	0.7 (2)	0.4 (9)	**1.7 (27)**	−6.6 (0)	−0.5 (2)	−1.1 (2)	−0.4 (4)	**1.5 (14)**	−6.6 (0)
Z2954	L-arabinose-binding periplasmic protein	1 (43)	1.4 (70)	−2.1 (2)	−0.3 (29)	1.2 (95)	**−8.4 (0)**	**−2.6 (2)**	**−4.8 (1)**	**−3.2 (3)**	1.3 (60)	**−8.4 (0)**
Z3425	PTS system fructose-specific transporter subunits IIBC	1 (3)	**1.8 (7)**	1.5 (1)	1.5 (8)	1.3 (7)	−5.4 (0)	−0.2 (1)	0.4 (3)	**5.9 (112)**	**3.2 (17)**	−5.4 (0)
Z3426	1-phosphofructokinase	1 (4)	0.0 (2)	−4.9 (0)	**2.1 (15)**	1.2 (9)	−4.9 (0)	0.3 (2)	1.6 (8)	**5.9 (139)**	**2.7 (16)**	−4.9 (0)
Z3427	bifunctional PTS system fructose-specific transporter subunit IIA/HPr protein	1 (2)	0.9 (2)	−4.3 (0)	1.2 (4)	−0.6 (1)	−4.3 (0)	**2.7 (5)**	1.3 (3)	**7.3 (192)**	**4.0 (21)**	−4.3 (0)
Z3511	UDP-4-amino-4-deoxy-L-arabinose--oxoglutarate aminotransferase	1 (122)	**−1.9 (19)**	**−2.2 (4)**	−0.4 (76)	−1.0 (58)	−0.8 (17)	**−2.9 (5)**	**−4.0 (5)**	**−4.8 (2)**	0.3 (87)	**−4.2 (1)**
Z3512	undecaprenyl phosphate 4-deoxy-4-formamido-L-arabinose transferase	1 (75)	**−2.5 (7)**	−0.5 (9)	−1.8 (18)	**−2.2 (15)**	−1.5 (7)	**−3.9 (2)**	**−5.5 (1)**	**−3.6 (3)**	−0.7 (27)	**−3.2 (1)**
Z3513	bifunctional UDP-glucuronic acid decarboxylase/UDP-4-amino-4-deoxy-L-arabinose formyltransferase	1 (88)	**−3.3 (5)**	−1.7 (4)	−1.3 (29)	**−2.2 (17)**	**−2.4 (4)**	**−3.2 (3)**	**−2.8 (7)**	**−4.3 (2)**	−0.4 (39)	**−4.5 (1)**
Z3515	4-amino-4-deoxy-L-arabinose transferase	1 (64)	−1.8 (11)	−1.6 (3)	0.7 (91)	−0.7 (39)	−1.8 (5)	−1.8 (6)	**−2.4 (8)**	**−3.0 (5)**	−0.4 (28)	**−9.7 (0)**
Z5648	phage shock protein G	1 (2)	**3.1 (11)**	**6.4 (31)**	**4.0 (29)**	**2.8 (14)**	**4.5 (13)**	2.1 (3)	**3.0 (11)**	2.2 (6)	**7.7 (257)**	**8.5 (140)**
Z5717	arginine:agmatin antiporter	1 (5)	0.2 (3)	−5.7 (0)	**−3.2 (0)**	1.3 (11)	−5.7 (0)	−0.5 (1)	**3.9 (46)**	**7.5 (528)**	**1.8 (9)**	−5.7 (0)
Z5719	biodegradative arginine decarboxylase	1 (3)	−0.1 (2)	0.3 (1)	−1.7 (1)	−1.4 (1)	−6.0 (0)	**−6.0 (0)**	0.3 (3)	**8.3 (647)**	1.2 (4)	−6.0 (0)
Z5734	lysine decarboxylase 1	1 (1)	1.6 (2)	−4.0 (0)	−0.1 (1)	0.6 (1)	−4.0 (0)	−4.0 (0)	−4.0 (0)	**7.6 (107)**	0.5 (1)	−4.0 (0)
Z5735	lysine/cadaverine antiporter	1 (0)	0.6 (0)	3.2 (1)	−2.1 (0)	0.6 (1)	−2.1 (0)	2.1 (1)	−2.1 (0)	**5.3 (10)**	−2.1 (0)	−2.1 (0)

^1^For each gene, the first number indicates the logFC of a certain condition compared to LB; RPKM values are shown in parentheses. Significantly differentially expressed genes are in bold (i.e., p values ≤ 0.05 in *edgeR*). Note that Z3511-Z3515 are not only active on sprouts, but also in LB medium. For a graphic version, indicating the magnitude of the absolute value of logFC in shades of grey, see Additional file 4.

#### Response to stress

We assign azoreductase *azoR* (Z2315, Table 5, Figure 5B-C) to the stress related genes. Azo dyes are a class of colorants used in chemical, pharmaceutical and food industries. They are carcinogenic and can cause severe environmental problems [80]. Bacterial azoreductases can reduce these dyes in a NAD(P)H dependent reaction [81]. However, azo dyes are human made compounds. The environmental role of azoreductase is unknown [82]. As we measured high levels of transcripts on sprouts (logFC = 4.1, RPKM = 190), we speculate on a role of this enzyme in detoxification of secondary plant metabolites directed against, or modulating, the bacterial microbiome. Indeed, Liu *et al.*[82] found that *azoR* protects *E. coli* against thiol-specific stresses caused by electrophilic quinones.

Up-regulation (logFC = 3.8) of aquaporin *aqpZ* (Z1109, Table 5) on radish sprouts may indicate hypoosmotic stress [83] since aquaporins are proteins conducting water (or glycerol), but only about one quarter of the bacterial species possess an *aqpZ* homolog. The role of *aqpZ* in osmotic regulation is under debate due to conflicting data (see [83] and references therein). However, Tanghe *et al.*[83] hypothesize that transport of other small uncharged molecules besides water may play a role associated with certain lifestyles or ecological niches.

A membrane stress response [84] of EHEC on sprouts is supported by the high activity of the phage shock genes *pspABC* and *pspG* (Z2479, Z2480, Z2482, Z5648, Table 5; [85]) with logFCs between 3 and 8 on radish sprouts, perhaps indicating that secondary plant metabolites secreted by the radish sprouts may impair membrane integrity. Further, we identified an up-regulated membrane protein (YhdV, Z4628) and a quercetinase homolog (YhhW, Z4807). The flavonoid quercetin is widely distributed in plants and potentially toxic. Thus, YhhW may be involved in its detoxification [46].

Another up-regulated gene (logFC of 7.5) indicative of a stress response is the acid shock protein precursor AsrA (Z2591, Table 5: [86]). This small protein localizes in the periplasm and is further processed to a 8 kDa fragment, which is the active form of this proposed chaperone [25]. It appears that low pH is only a necessary, but not a sufficient condition, to induce *asrA* as it is not active in acidified LB-nitrite. In addition, osmotic stress also induces *asrA*[25, 87, 88].

Finally, *narU* (Z2243) encodes a protein forming a single channel for nitrate uptake and nitrite extrusion [89]. It is strongly up-regulated (logFC of 7.0) on radish sprouts and only to a logFC of 4.4 in LB-nitrite.

#### Adhesion to the plant surface

Curli fiber genes are associated with adhesion to plants (e.g., [90]). These fimbriae-like structures are a major factor for the formation of biofilms and adhesion to surfaces [91]. The highest activity of these six genes *csgGFEDBA* (Z1670 – Z1676) was determined on radish sprouts (Table 5). An additional indicator for adhesion to radish sprouts is the up-regulation of *bssS* (Z1697, Table 5), a regulatory gene for biofilm formation [92]. The increased transcription level of curli-related genes together with *bssS* corroborates the hypothesis of Fink *et al*. [47] that lettuce leaves are colonized by using curli fibers and by fine tuning biofilm formation.

**Figure 6:**
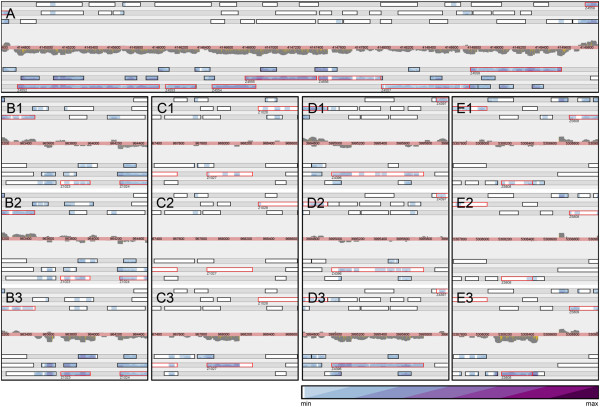
**Visualization of the sequencing reads (= transcription) using the NGS overlap searcher** [93]. The tool shows a plot of the read coverage in the middle. Forward strand reads are plotted above, reverse strand reads below the center line. The read starts are indicated in yellow. The bars above (forward strand) and below (reverse strand) the middle bar show all ORFs ≥ 90 nt in the six different reading frames. Annotated ORFs are in red. The tool shows the coverage also in the ORF bars according to the scaling in the lower right corner. **A:** selection of an “empty” region of the genome on the forward strand (4144776 – 4149762). The coverage shown is a sum signal of all eleven conditions sequenced on the SOLiD system. Only eight reads are found on the forward strand of this region. **B1-B3:** example for a regulated hypothetical gene, Z1023 (see Table 2), in LB medium (B1), minimal medium (B2), and on radish sprouts (B3). **C1-C3:** regulated hypothetical gene Z1027 (see Table 2), in LB medium (C1), minimal medium (C2), and on radish sprouts (C3). **D1-D3:** regulated hypothetical gene Z4396 (Table 2), in LB medium (D1), minimal medium (D2), and on radish sprouts (D3). **E1-E3:** regulated hypothetical gene Z5808 (see Table 2), in LB medium (E1), minimal medium (E2), and on radish sprouts (E3). The color map values range from 0.1 to 3 × 10^5^, the exact expression values for each gene are listed in Table 2.

We identified nine hypothetical genes in radish sprouts with a logFC higher than 5, which are only active on sprouts (summarized in Table 2 and visualized in Figure 6B-E). One of those, *yjfY*, was already found induced on lettuce leaves [47]. This gene is also active in biofilm growth [48, 49]. We found additional hypotheticals that play a role in biofilm formation, which are summarized in Table 2 including references for them.

#### Radish sprouts as a reservoir of EHEC?

Sprouts were inoculated with 4 × 10^2^ cfu/g plant EHEC and grown for several days. The growth curve in Figure 5A illustrates that EHEC grows very well on the plants, reaching 2 × 10^7^ cfu/g plant, apparently without affecting the plant phenotype. As shown above, EHEC expresses many unique genes when it thrives on the plant surface, including adhesin, membrane proteins, transport proteins, metabolic proteins and a variety of stress response proteins. We conclude that radish sprouts are a suitable habitat for EHEC to proliferate. However, this experiment reflects a mono-association of EHEC and radish sprouts and, therefore, does not yet allow a conclusion whether plants in general serve as a natural reservoir of EHEC.

#### EHEC as “vegetarian”?

Obviously, EHEC is able to survive and proliferate on and in plants. This has now been shown several times by different groups (e.g. [94]). However, after EHEC had been described as a pathogen in 1982, it was dubbed “hamburger disease”, since many outbreaks were related to undercooked minced meat. For quite some time more or less the only reservoir considered for pathogenic enterobacteria was meat, milk, and products thereof [95]. However, in hindsight, a possible “vegetarian” life style of EHEC should have been considered years ago, since EHEC contains genes to metabolize different sugars (some of which exclusively produced by plants): *fruAKB* for fructose, *otsAB* for trehalose, and *araAHGF* for arabinose. Using BLAST, we found that plant pathogens or plant associated genera, such as *Ralstonia*, *Xanthomonas*, *Erwinia*, *Rhizobium*, and *Dickeya* also contain such operons. Next, EHEC forms biofilms on plant surfaces using curli. Again, species of *Rahnella* and *Serratia* contain *csgA*. The quercetinase homolog *yhhW* found in EHEC is also present in *Pectobacterium carotovorum*, and *Serratia proteamaculans*. Stress related EHEC-genes induced while growing on sprouts, such as *asr* and *pspABCG,* are found in *Burkholderia gladioli*, and *Pectobacterium* species. Finally, as shown in this paper, *azoR* is induced in EHEC when growing on radish and *azoR*-homologs are found in species of *Serratia*, *Erwinia*, *Pectobacterium*, and *Dickeya*. Taking together, it would be quite interesting to scan the EHEC-genome for homologous genes from other bacteria, which are known to be induced in the respective niche of each bacterium and to see, if EHEC can thrive in this niche as well and which genes are induced. Strand-specific transcriptomes supply an excellent technique to substantiate such hypotheses.

## Conclusions

Distinguishing weakly transcribed genes from background transcription is a general problem in NGS transcriptomics. Our proposed statistical method is based on the data of the actual experiment, thus also takes the sequencing depth into account. Genes are classified into “active” or “inactive”, based on a sound statistical evaluation and not on arbitrarily chosen threshold values of reads or RPKM. We sequenced biological replicates of transcriptomes using the SOLiD and the Illumina system and showed a high correlation between both approaches, confirming that the SOLiD and Illumina system produce equivalent data. This is interesting insofar as PCR-artifacts and other biased reactions during library preparation are a possible source of the uneven coverage of a given gene with reads. However, when comparing relative transcription (hence, regulation), these effects apparently tend to cancel each other out. Otherwise, there would be no or only weak correlation between data gained with the SOLiD and the Illumina system.

We discovered a unique set of active genes for each condition tested and, remarkably, most genes of EHEC appear to be active under at least one condition. Indeed, under environmental conditions more hypothetical genes were found to be active than in standard lab media. This is not too surprising, since growth of *E. coli* in standard medium has been examined over and over again. Interestingly, only a minority of genes (2.7%) were not active under any condition tested by us. We therefore suggest that the general assumption that large numbers of genes are over-annotated in bacterial genomes may be wrong. In addition, such genes might be active in habitats not yet probed. Finally, *azoR* exemplarily shows that transcriptome profiling still is and will be a powerful technique to find new roles for genes. *azoR* was formerly only known to destroy artificial azo-dyes, but its high induction on plants suggests a detoxification role in nature. This finding provides an entry point to test natural plant substances for *azoR* induction and to observe growth (impairments?) of an Δ*azoR* mutant to further elucidate the behavior of EHEC and other pathogens in nature. Similarly, other highly induced or repressed genes are now new candidates for a detailed functional description.

## Methods

### Strains and culture conditions

If not stated otherwise, *E. coli* O157:H7 EDL933 (EHEC) (Collection de l’Institute Pasteur: CIP 106327) was incubated in liquid medium at 37°C with shaking (180 rpm) by adding 1 ml overnight culture (about 10^9^ cfu) to 100 ml medium. Growth curves were measured either by optical density (OD_600nm_) or counting colony forming units (cfu) after serial platings. Before harvesting, samples were plated on CHROMagar O157 (CHROMagar, France) to confirm identity. In all cases, bacterial cells were harvested at the transition from late exponential to early stationary phase by centrifugation (20,000 × *g*, 1°C, 3 min) and frozen in liquid nitrogen for storage.

LB: Tenfold diluted lysogeny broth was used as reference medium. Cells were harvested after 3.5 h at about 3.1 × 10^8^ cfu/ml.

LB-15°C: Transcription was determined at 15°C in tenfold diluted LB medium and harvested at 3.1 × 10^8^ cfu/ml.

MM: M9 minimal medium was prepared as described [96] and cells harvested after 12 h at about 2.5 × 10^9^ cfu/ml.

LB-pH9: Tenfold diluted LB medium at alkaline pH was buffered with 10 mM CHES and the pH was adjusted to 9.0 at 37°C and was filter sterilized. After 7 h, the cells reached 1.5 × 10^8^ cfu/ml and were harvested.

LB-pH4: Tenfold diluted LB medium at pH4 was adjusted to 4.0 at 37°C and filter sterilized. Cells were harvested at 2.0 × 10^8^ cfu/ml.

LB-nitrite: For nitrite, we added 200 mg/L sodium nitrite to 10-fold diluted LB and adjusted it to pH6. Harvest was after 6.5 h at 2.9 × 10^8^ cfu/ml.

Spinach: For spinach medium, whole spinach leaves were homogenized (Agienda Agricola Pistelle, Kaufland, Germany) on ice using an Ultraturrax D50. The mush was centrifuged (1 h, 30,000 × *g*, 5°C), decanted, filtered (2.5 μm pore size), centrifuged (2 h, 30,000 × *g*, 5°C), decanted and sterile filtered (0.2 μm). After 5 h of growth, we harvested the cells at 6.0 × 10^8^ cfu/ml.

LB-antibiotics: Tenfold diluted LB was supplemented with 2 μg/ml sulfamethoxazole and 0.4 μg/ml trimethoprim. This medium was inoculated with 2 ml of overnight culture. Cells cannot divide anymore in this medium and the increase in OD_600nm_ is due to massive cell elongation. We harvested the cells at the peak of OD_600nm_ at 0.194.

LB-solid: For growth on solid medium, about 500 colonies were grown on undiluted LB agar plates and harvested after 17 h at 37°C. Colonies were transferred directly to Trizol (see below) for RNA extraction.

Sprouts: Radish sprout seeds were sterilized (5 min 70% ethanol, 10 min 1% NaOCl with 0.1% Tween), then washed five times with sterile water and subsequently incubated in sterile MS medium without glucose [97] in sterile plastic boxes (1 L total volume, passively aerated). After germination, seedlings were tested for sterility by plating a sample on LB agar. After 5 days of growth, the shoots were inoculated 10 min with 1 L ¼-concentrated Ringer solution containing 10^3^ cfu/ml EHEC. The superfluous medium was decanted and cfu/g was periodically determined as follows: infected shoots were washed and bacterial numbers of the washing liquid were determined by serial dilution platings. After 120 hours, the transition from exponential to stationary phase could be determined (see Figure 5A). Bacteria were harvested by gently shaking the seedlings in cold ¼-concentrated Ringer (+1% Tween-20; 4°C) for 1 min. Bacteria were collected by centrifugation from the decanted Ringer as above.

Cattle feces: The number of cultivatable bacteria of cattle feces was determined by serial platings on LB-agar plates after 12 h at 37°C. The cattle feces were subsequently inoculated with 1000-fold number of EHEC, pre-grown in 1 L LB to stationary phase. When the bacteria had reached stationary phase, they were harvested by centrifugation and re-suspended in 7 ml ¼-concentrated Ringer. We added this suspension to 10 g of cattle feces and mixed it thoroughly. After 6 h at 37°C, bacterial cells were harvested by adding 90 ml cold ¼-concentrated Ringer shaking for 10 s, sedimentation for 30 s, decanting, and centrifugation.

### RNA isolation and propagation

RNA was isolated with Trizol (Invitrogen, USA). One ml Trizol and about 200 μl of 0.1 mm zirconia beads were added to 50 μl cell pellet. The cells were disrupted by bead-beating (FastPrep-24, MP Biomedicals, USA), thrice for 45 s at 6.5 m/s, and cooled for 5 min on ice in between. Subsequently, the Trizol-manual was followed and the RNA-pellet was dissolved in RNase free water. Since 90-95% of the total RNA consists of ribosomal RNA [30], we applied the Ribominus Transcriptome Isolation Kit (Yeast and Bacteria, Invitrogen, USA). The manufacturer’s manual was followed but the RNA was co-precipitated with 1 μl glycogen, using 2.5 volumes 100% ethanol and 0.1 volumes 3 M sodium acetate, instead of the concentration modules included. Residual DNA was removed with the TURBO DNA-*free* Kit (Applied Biosystems, USA).

### Whole transcriptome RNA library preparation – SOLiD system

Fragmentation, hybridization, ligation, reverse transcription of enriched total RNA and amplification of the cDNA was carried out using the SOLiD Total RNA-seq Kit (Applied Biosystems, USA). Briefly, RNA was fragmented with RNase III for 9 min. We purified the reaction mixture with the miRNeasy Mini Kit (Qiagen, Germany). This returns high amounts of RNA and removes proteins from the RNase treatment. Hybridization and ligation was performed using the SOLiD Adaptor Mix at 65°C for 10 min and the Ligation Enzyme Mix at 16°C for 16 h following the manufacturer’s instructions. The ligation reaction was directly added to the RT reaction mix containing SOLiD RT Primer and ArrayScript Reverse Transcriptase. The mixture was incubated at 42°C for 30 min. After purification using the MinElute® PCR Purification Kit (Qiagen, Germany), the cDNA was size selected for 150–250 nt cDNA with Novex® 6% TBE-Urea Gels. The selected cDNA was directly amplified from the gel in 15 PCR cycles. Here, we used the SOLiD Transcriptome Mutiplexing Kit. SOLiD 3′ PCR primers were replaced by different barcoded SOLiD 3′ PCR primers for different conditions. Two libraries, spinach and LB-nitrite, were split before and further treated independently to obtain technical replicates. The amplified DNA was purified with the PureLink PCR Micro Kit (Invitrogen, USA). The amounts of RNA/DNA were measured with a NanoDrop spectrophotometer. The quality and size distribution of the isolated and depleted RNA was assessed on the Agilent 2100 Bioanalyzer with Agilent DNA 1000 Kit and RNA 6000 Pico Kit. SOLiD System templated bead preparation and sequencing on the SOLiD 4.0 system was conducted by CeGaT GmbH (Tübingen, Germany).

### Whole transcriptome RNA library preparation – Illumina system

Biological replicates of LB medium and radish sprouts were sequenced on an Illumina MiSeq sequencer. One μg RNA was fragmented as described in Flaherty *et al.*[98] using a Covaris sonicator and the RNA-fragments precipitated with glycogen and 2.5 volumes 100% ethanol. RNA fragments were dephosphorylated using Antarctic phosphatase (10 units per 300 ng RNA, supplemented with 10 units Superase, 37°C for 30 min). The fragments were recovered using the miRNeasy Mini Kit (Qiagen, Germany). Subsequent phosphorylation was carried out using 20 units T4 polynucleotide kinase, supplemented with 10 units RNase inhibitor Superase (Life Technologies, USA) at 37°C for 60 min, and recovered using the miRNeasy Mini Kit. The prepared RNA was processed further with the TruSeq Small RNA Sample Preparation Kit (Illumina, USA): The whole sample was concentrated in a Speedvac (Eppendorf, Germany) at 30°C for 1 hour to 5 μl final volume. The RNA 3′ and 5′ adapters were ligated to the fragments strand specifically. The ligated fragments were reverse transcribed using the SuperScript II Reverse Transcriptase kit (Life Technologies, USA). The subsequent PCR reaction was run in 11 cycles at an annealing temperature of 60°C. Amplified cDNA was purified on 6% Novex TBE polyacrylamide gels. For this, each complete sample was loaded into three wells. The gel was run for 45 minutes at 145 V in Novex TBE buffer. Afterwards, the DNA was stained with SYBR Gold. Fragments were size selected between 190 and 300 base pairs according to the ladder. The chosen length corresponds to an insert length of 50 to 100 base pairs. The gel pieces were transferred to a pierced 0.5 ml micro-centrifuge tube, placed in a 1.5 ml tube and centrifuged at 13,000 × *g* for 5 min at room temperature. The gel debris was eluted in 300 μl ddH_2_O for three hours under intense rotation. The eluate was filtered in a 0.22 μm Spin-X spin filter (Corning, USA) and the debris was discarded. The solution was ethanol precipitated with glycogen and sodium acetate and re-suspended in 10 μl elution buffer. The library was quantified using a Qubit (Life Technologies, USA), and denatured in 0.1 N NaOH. Next, it was diluted with the supplied HT1 buffer to an end concentration of 8 pM. The sequencing was conducted on a MiSeq sequencer with 50 cycles of library sequencing.

### Bioinformatics

SOLiD output as QUAL and CSFASTA files was converted to FASTQ with Galaxy [99, 100]. We mapped SOLiD and Illumina FASTQ files to the reference genome of EHEC [GenBank:NC_002655] and to the plasmid pO157 [GenBank:NC_007414] using Bowtie [101] (settings for SOLiD data: 28 nt seed length, maximal two mismatches in the seed, a maximal threshold of 70 for the sum of the quality values at mismatched positions; Illumina data: 20 nt seed length, 0 mismatches in the seed) implemented in Galaxy. Using Samtools output SAM files were filtered for mappable reads only [102]. We further converted SAM files to BAM files and indexed them to create BAM.BAI files. The data were visualized with BamView [103] implemented in Artemis 13.0 [104]. Raw data have been uploaded to the Gene Expression Omnibus [GEO:GSE48199].

### Normalizing to RPKM values

The number of reads were normalized to reads per kilobase per million mapped reads (RPKM; [29]). Using this method, the number of reads is normalized with respect to the sequencing depth and the length of a given gene. For determination of counts and RPKM values, BAM files were imported into *R*[105] using *Rsamtools*[106]. For further processing, the *Bioconductor*[107] packages *GenomicRanges*[108] and *IRanges*[109] were used. Gene locations were determined by *RefSeq*[110] and *GenBank*[111] PTT files. The locations of the 16S rRNA and 23S rRNA are given by the RNT file from *RefSeq*. The method countOverlaps of *IRanges*[109] was used to determine the remaining reads overlapping a 16S or 23S rRNA gene. We discarded these reads from further analysis due to the artificial removal of these rRNAs using the Ribominus kit as described above. countOverlaps is also used to determine the number of reads overlapping a gene on the same strand (counts). With these counts we generated the RPKM values. For the value “million mapped reads”, the number of reads mapped to the genome, minus the reads overlapping a 16S or 23S rRNA gene, were used (see above). The differential gene expression was analyzed with the *Bioconductor* package *edgeR* (version 3.2.3) using the counts [112].

### Differential expression analysis

The *Bioconductor* package *edgeR* uses an overdispersed Poisson model to estimate biological variability. Such empirical Bayes methods diminish variances across the genes [112]. The dispersion of the data was analyzed by sequencing biological replicates using two different NGS platforms (SOLiD and Illumina) of the LB reference medium and the radish sprouts condition. Confirming by statistical analysis that both sequencing platforms showed the same results for the biological replicates (see Results and Discussion), data of the experiments were merged. We present the data as a log_2_-fold change (logFC) of a gene in each condition compared to LB medium as basis. log_2_ was chosen since the cDNA is amplified using the non-linear process of a PCR-reaction in which, in first approximation, the number of fragments grows exponentially with each cycle. In the result tables values in parentheses are RPKM values.

### Determination of background transcription

Incidence for the transcription of a gene is given by a transcription level higher than a supposedly random transcription. This pervasive transcription distributes all over the genome, also in non-coding regions (e.g., [31]). We determined random transcription by manually selecting regions of the genome that are obviously free of annotated genes. Some regions are antisense to annotated genes. We analyzed these regions also visually for the absence of non-coding RNAs or any other conspicuous transcription patterns. Figure 6A shows an example screenshot of one region. The genome positions (matching to [GenBank:NC_002655]) of the regions used are: complement(264387 – 269904), c(430056 – 435429), c(524890 – 530056), c(613235 – 620336), 2293616 – 2309141, 3707862 – 3711921, 3840351 – 3844419, 4121574 – 4126839, 4144776 – 4149762, 4298037 – 4302222, 4494846 – 4501272, 4615115 – 4619372, c(4635078 – 4639956), 5199469 – 5210215, 5263170 – 5266662, c(5277831 – 5281854), c(5282151 – 5286750), and c(5294994 – 5299602). Taken together all regions comprise a virtually “empty” part of the genome of 104.192 base pairs in length (~2%), which is supposed to be randomly transcribed only. We calculated the RPKM value for these parts for every condition in the same manner as for the annotated genes. Genes were defined as being active or turned “on” if the probability that the signal is due to the background is significantly low (p ≤ 0.05). We consider a gene as silent if it is not covered by a read in any of the conditions.

### Statistical analysis of active genes

Reads observed over a gene may be solely attributed due to the background noise or background transcription (see Results and Discussion). Therefore we employ a background model as explained in the following. We assume that on average a *background* read will start at a position *i* with a given rate λ per base, and that the starts of background reads are mutually independent. Hence, a reasonable model for the read starts is a Poisson process with rate λ (see, e.g., [113]). Suppose we observe *m* reads over a gene of length *g*. The P-value of the hypothesis that the reads are solely due to the background is then given by1

with

Equation (1) can be numerically evaluated given the gene length and the corresponding λ. To estimate the parameter λ we used the data of all regions with no transcription (see above) separately for each experimental condition (Additional file 6: Table S1).

### Heat map generation

The generation of heat maps allows analysis of the data for similar global response patterns. We visualized the logFC values of all conditions sequenced on the SOLiD system including LB medium as reference with heat maps using the R [105] method heatmap.2 of the package *gplots*[114]. Hierarchical complete linkage clustering was applied to rows and columns with Euclidean distance as distance measure. The used color map was linearly interpolated in RGB with the colorRampPalette method of R from the *RColorBrewer*[115] color palette RdBu with eleven colors.

### Availability of supporting data

The RNA-seq raw data were deposited to NCBI GEO with the accession number GSE48199 (http://www.ncbi.nlm.nih.gov/geo/query/acc.cgi?acc=GSE48199).

## Electronic supplementary material

Additional file 1: Table S2: Probability of each gene under each condition, whether its reads result from background or from an activity above background. (XLSX 336 KB)

Additional file 2: Table S3: Silent genes, transcriptionally inactive silent genes. (DOCX 21 KB)

Additional file 3: Table S4: The transcriptional regulation of all 5379 protein-coding genes for the genome and plasmid for each condition. (XLSX 703 KB)

Additional file 4: **Graphic versions of Tables** 2 **,** 3 **,** 4 **, and** 5 . The magnitude of the absolute value of logFC is indicated by shades of grey. (DOCX 98 KB)

Additional file 5: Table S5: Transcriptional regulation of the virulence associated genes. (DOCX 32 KB)

Additional file 6: Table S1: Regions of the genome with background transcription only for each condition. (XLSX 12 KB)
