# Supplementary material for: Comparison of strand-specific transcriptomes of enterohemorrhagic Escherichia coli O157:H7 EDL933 (EHEC) under eleven different environmental conditions including radish sprouts and cattle feces
Source: BMC Genomics. 2014 May 9;15:353. doi: 10.1186/1471-2164-15-353 (PMC4048457; doi:10.1186/1471-2164-15-353)
Supplement: Supplementary file 2 — Additional file 2: Table S3: Silent genes, transcriptionally inactive silent genes. (DOCX 21 KB) [file 12864_2013_6096_MOESM2_ESM.docx]

**Table S3. Silent genes.** None of the 144 genes listed is covered by a read in any of the eleven sequenced conditions. Note that 69.4% (100 genes) of these genes are annotated as hypothetical.

| gene tag | product | gene tag | product | gene tag | product | gene tag | product | gene tag | product |
| --- | --- | --- | --- | --- | --- | --- | --- | --- | --- |
| Z0021 | hypothetical protein | Z1430 | hypothetical protein | Z1817 | hypothetical protein | Z2393 | hypothetical protein | Z3151 | nicotinate-nucleotide--dimethylbenzimidazole phosphoribosyltransferase |
| Z0039 | hypothetical protein | Z1431 | hypothetical protein | Z1818 | putative antirepressor protein encoded by prophage CP-933N | Z2394 | hypothetical protein | Z3152 | cobalamin synthase |
| Z0341 | hypothetical protein | Z1456 | hypothetical protein | Z1821 | hypothetical protein | Z2396 | putative replication protein | Z3153 | adenosylcobinamide kinase/adenosylcobinamide-phosphate guanylyltransferase |
| Z0757 | triphosphoribosyl-dephospho-CoA synthase | Z1489 | putative outer membrane protein Lom precursor of bacteriophage BP-933W | Z1822 | hypothetical protein | Z2397 | hypothetical protein | Z3159 | putative outer membrane receptor for iron compound or colicin |
| Z0760 | citrate lyase beta chain (acyl lyase subunit) | Z1532 | hypothetical protein | Z1823 | hypothetical protein | Z2398 | hypothetical protein | Z3226 | tyrosine phosphatase |
| Z0761 | citrate lyase subunit gamma | Z1540 | hypothetical protein | Z1824 | hypothetical protein | Z2399 | putative regulatory protein Cro of prophage CP-933R | Z3278 | putative chaperone protein |
| Z0848 | rhsC protein in rhs element | Z1561 | hypothetical protein | Z1825 | insertion element IS2 transposase InsD | Z2400 | partial putative represssor protein encoded within prophage CP-933R | Z3327 | hypothetical protein |
| Z0849 | hypothetical protein | Z1566 | hypothetical protein | Z1826 | putative IS encoded protein | Z2402 | hypothetical protein | Z3334 | hypothetical protein |
| Z0870 | hypothetical protein | Z1577 | hypothetical protein | Z1829 | hypothetical protein | Z2403 | hypothetical protein | Z3347 | hypothetical protein |
| Z0896 | hypothetical protein | Z1596 | hypothetical protein | Z1867 | putative integrase of prophage CP-933X | Z2404 | prophage CP-933R superinfection exclusion protein | Z3388 | hypothetical protein |
| Z0962 | hypothetical protein | Z1618 | hypothetical protein | Z1901 | hypothetical protein | Z2406 | FtsZ inhibitor protein | Z3599 | putative fimbrial chaperone |
| Z1018 | hypothetical protein | Z1619 | hypothetical protein | Z1903 | hypothetical protein | Z2408 | hypothetical protein | Z3745 | hydrogenase 4 membrane subunit |
| Z1038 | hypothetical protein | Z1621 | hypothetical protein | Z1919 | hypothetical protein | Z2409 | exonuclease VIII | Z3767 | hypothetical protein |
| Z1122 | hypothetical protein | Z1764 | partial integrase for prophage CP-933N | Z1967 | hypothetical protein | Z2410 | recombination and repair protein RecT | Z3938 | hypothetical protein |
| Z1127 | hypothetical protein | Z1765 | putative excisionase for prophage CP-933N | Z2052 | hypothetical protein | Z2412 | restriction alleviation and modification protein | Z4034 | hypothetical protein |
| Z1156 | hypothetical protein | Z1768 | hypothetical protein | Z2123 | hypothetical protein | Z2413 | hypothetical protein | Z4042 | hypothetical protein |
| Z1157 | hypothetical protein | Z1770 | hypothetical protein | Z2240 | hypothetical protein | Z2414 | hypothetical protein | Z4090 | hypothetical protein |
| Z1179 | hypothetical protein | Z1771 | hypothetical protein | Z2263 | Rhs element protein | Z2415 | putative integrase for prophage CP-933R | Z4177 | hypothetical protein |
| Z1180 | hypothetical protein | Z1776 | hypothetical protein | Z2293 | hypothetical protein | Z2465 | putative beta-phosphoglucomutase | Z4320 | hypothetical protein |
| Z1182 | hypothetical protein | Z1777 | hypothetical protein | Z2325 | hypothetical protein | Z2947 | hypothetical protein | Z4327 | hypothetical protein |
| Z1186 | hypothetical protein | Z1779 | hypothetical protein | Z2342 | partial putative outer membrane protein Lom precursor encoded by prophage CP-933R | Z3137 | hypothetical protein | Z4748 | hypothetical protein |
| Z1210 | putative histone | Z1780 | hypothetical protein | Z2348 | partial putative phage tail protein encoded by prophage CP-933R | Z3138 | shikimate transporter | Z5015 | hypothetical protein |
| Z1345 | antitermination protein Q homolog of cryptic prophage CP-933M | Z1797 | putative antirepressor of prophage CP-933N | Z2385 | hypothetical protein | Z3139 | AMP nucleosidase | Z5027 | hypothetical protein |
| Z1346 | hypothetical protein | Z1799 | partial tonB-like membrane protein encoded within prophage CP-933N | Z2386 | hypothetical protein | Z3140 | hypothetical protein | Z5226 | hypothetical protein |
| Z1363 | hypothetical protein | Z1800 | hypothetical protein | Z2387 | hypothetical protein | Z3143 | hypothetical protein | Z5430 | hypothetical protein |
| Z1364 | hypothetical protein | Z1808 | hypothetical protein | Z2389 | putative DNA modification methyltransferase encoded within prophage CP-933R | Z3144 | hypothetical protein | Z5443 | putative fructose-like phosphotransferase system subunit EIIA |
| Z1373 | hypothetical protein | Z1809 | hypothetical protein | Z2390 | hypothetical protein | Z3146 | transcriptional regulator Cbl | Z5446 | rhamnulose-1-phosphate aldolase |
| Z1374 | hypothetical protein | Z1810 | hypothetical protein | Z2391 | hypothetical protein | Z3147 | nitrogen assimilation transcriptional regulator | Z6011 | hypothetical protein |
| Z1376 | putative tail component encoded by cryptic prophage CP-933M; partial | Z1816 | hypothetical protein | Z2392 | hypothetical protein | Z3150 | hypothetical protein |  |  |
