# Supplementary material for: Comparison of strand-specific transcriptomes of enterohemorrhagic Escherichia coli O157:H7 EDL933 (EHEC) under eleven different environmental conditions including radish sprouts and cattle feces
Source: BMC Genomics. 2014 May 9;15:353. doi: 10.1186/1471-2164-15-353 (PMC4048457; doi:10.1186/1471-2164-15-353)
Supplement: Supplementary file 4 — Additional file 4: Graphic versions of Tables 2 , 3 , 4 , and 5 . The magnitude of the absolute value of logFC is indicated by shades of grey. (DOCX 98 KB) [file 12864_2013_6096_MOESM4_ESM.docx]

Table 2 Hypothetical genes with a logFC ≥ 5 in transcription levels in a single condition compared to LB. For each gene, the first number indicates the logFC of a certain condition compared to LB; RPKM values are shown in parentheses. The magnitude of the absolute value of logFC is indicated by shades of grey. Significantly differentially expressed genes are in bold (i.e., p values ≤ 0.05 in *edgeR*).

| gene tag | product | Ref’s |  | LB | LB-pH9 |  | LB-pH4 |  | LB-15°C |  | LB-nitrite |  | LB-antibiotics |  | LB-solid |  | minimal medium |  | spinach juice |  | radish sprouts |  | faeces |  |
| --- | --- | --- | --- | --- | --- | --- | --- | --- | --- | --- | --- | --- | --- | --- | --- | --- | --- | --- | --- | --- | --- | --- | --- | --- |
| Z0840 | hypothetical protein |  | LB-pH4 | 1 (2) | 1.4 (3) |  | **5.5 (18)** |  | 2.0 (8) |  | -1.4 (0) |  | -1.4 (0) |  | -1.4 (0) |  | -1.4 (0) |  | 2.9 (10) |  | -1.4 (0) |  | **4.4 (9)** |  |
| Z1576 | hypothetical protein |  |  | 1 (0) | 0.0 (0) |  | **5.3 (3)** |  | **3.4 (4)** |  | 0.0 (0) |  | 0.0 (0) |  | 0.0 (0) |  | 0.0 (0) |  | 0.0 (0) |  | 3.0 (2) |  | 0.0 (0) |  |
| Z1850 | hypothetical protein |  |  | 1 (0) | 0.0 (0) |  | **5.3 (3)** |  | 0.0 (0) |  | 0.0 (0) |  | 0.0 (0) |  | 0.0 (0) |  | 0.0 (0) |  | 0.0 (0) |  | 0.0 (0) |  | 0.0 (0) |  |
| Z4062 | hypothetical protein |  | LB-15°C | 1 (9) | **2.7 (36)** |  | -4.4 (0) |  | **5.1 (275)** |  | 1.5 (26) |  | -4.4 (0) |  | 1.3 (8) |  | -4.4 (0) |  | 0.4 (7) |  | 0.1 (5) |  | -4.4 (0) |  |
| Z4925 | hypothetical protein |  |  | 1 (114) | **2.4 (388)** |  | 0.2 (23) |  | **5.0 (3365)** |  | **3.3 (1157)** |  | **-9.0 (0)** |  | -1.1 (18) |  | **2.7 (482)** |  | **4.1 (1163)** |  | **1.6 (210)** |  | -0.1 (18) |  |
| Z5688 | hypothetical protein |  |  | 1 (2) | 1.7 (5) |  | -3.6 (0) |  | **5.5 (90)** |  | **2.2 (10)** |  | -3.6 (0) |  | -3.6 (0) |  | 0.0 (1) |  | 1.2 (3) |  | **2.2 (6)** |  | -3.6 (0) |  |
| Z1924 | hypothetical protein |  | LB-nitrite | 1 (9) | **5.3 (250)** |  | **3.7 (23)** |  | **4.3 (182)** |  | **6.0 (659)** |  | -3.9 (0) |  | **4.7 (90)** |  | **3.2 (59)** |  | 1.6 (18) |  | **4.1 (101)** |  | **4.7 (47)** |  |
| Z0314 | prophage CP-933H, tail fiber |  |  | 1 (5) | -0.3 (2) |  | 0.4 (1) |  | 0.4 (6) |  | -0.7 (3) |  | **6.0 (92)** |  | -0.7 (1) |  | -0.3 (3) |  | -0.6 (2) |  | **-4.9 (0)** |  | -4.9 (0) |  |
| Z0316 | prophage CP-933H, tail fiber |  |  | 1 (12) | -0.6 (4) |  | -0.5 (1) |  | -0.1 (9) |  | 0.6 (17) |  | **5.3 (123)** |  | 0.4 (5) |  | -1.2 (3) |  | -0.6 (5) |  | **-2.8 (1)** |  | 0.1 (2) |  |
| Z0344 | hypothetical protein |  |  | 1 (1) | 1.3 (2) |  | 3.9 (3) |  | 1.1 (2) |  | 2.2 (5) |  | **5.2 (12)** |  | -1.4 (0) |  | -1.4 (0) |  | 2.8 (5) |  | 1.6 (2) |  | -1.4 (0) |  |
| Z0392 | hypothetical protein |  |  | 1 (0) | 2.8 (1) |  | 0.0 (0) |  | 0.0 (0) |  | 0.0 (0) |  | **5.6 (3)** |  | 0.0 (0) |  | **3.6 (2)** |  | 0.0 (0) |  | 0.0 (0) |  | 0.0 (0) |  |
| Z0949 | prophage CP-933K | [37] |  | 1 (17) | 0.5 (14) |  | **2.4 (15)** |  | 0.2 (17) |  | -0.7 (10) |  | **5.0 (143)** |  | -4.9 (0) |  | 0.3 (13) |  | -1.5 (3) |  | 0.3 (12) |  | **2.6 (17)** |  |
| Z1098 | hypothetical protein |  |  | 1 (1) | -2.1 (0) |  | -2.1 (0) |  | 1.8 (3) |  | 1.5 (3) |  | **5.1 (11)** |  | -2.1 (0) |  | 1.5 (2) |  | -2.1 (0) |  | 0.9 (1) |  | -2.1 (0) |  |
| Z1433 | prophage BP-933W | [37] |  | 1 (15) | 0.5 (13) |  | -5.1 (0) |  | 0.8 (22) |  | 1.0 (29) |  | **5.9 (231)** |  | -5.1 (0) |  | 0.0 (10) |  | -0.8 (5) |  | 0.3 (11) |  | 0.8 (4) |  |
| Z1434 | prophage BP-933W | [37, 38] |  | 1 (5) | 2.2 (14) |  | 2.4 (4) |  | **2.3 (21)** |  | 2.2 (21) |  | **7.2 (203)** |  | 1.3 (4) |  | **2.6 (19)** |  | 0.4 (3) |  | 1.6 (8) |  | -3.0 (0) |  |
| Z1441 | prophage BP-933W | [37] |  | 1 (274) | 0.0 (167) |  | -0.3 (35) |  | 1.4 (607) |  | 0.9 (464) |  | **5.5 (3123)** |  | -0.8 (52) |  | 1.3 (424) |  | 0.0 (160) |  | **1.6 (482)** |  | **-2.5 (8)** |  |
| Z1501 | hypothetical protein |  |  | 1 (27) | -0.1 (15) |  | -0.3 (4) |  | 0.0 (23) |  | -0.1 (24) |  | **5.1 (235)** |  | -0.1 (8) |  | 0.1 (18) |  | -0.7 (10) |  | 1.3 (38) |  | -0.7 (3) |  |
| Z1656 | hypothetical protein |  | LB-antibiotics | 1 (0) | -1.4 (0) |  | -1.4 (0) |  | -1.4 (0) |  | -1.4 (0) |  | **5.2 (7)** |  | -1.4 (0) |  | -1.4 (0) |  | -1.4 (0) |  | 1.6 (1) |  | -1.4 (0) |  |
| Z1840 | hypothetical protein |  |  | 1 (0) | 0.0 (0) |  | 0.0 (0) |  | 0.0 (0) |  | 2.7 (4) |  | **5.6 (9)** |  | 0.0 (0) |  | 0.0 (0) |  | 3.3 (4) |  | 0.0 (0) |  | 0.0 (0) |  |
| Z3353 | hypothetical protein |  |  | 1 (0) | 0.0 (0) |  | 0.0 (0) |  | 0.0 (0) |  | 0.0 (0) |  | **7.9 (29)** |  | 0.0 (0) |  | 0.0 (0) |  | 0.0 (0) |  | 3.0 (2) |  | 0.0 (0) |  |
| Z3369 | prophage CP-933V | [37] |  | 1 (7) | -0.3 (3) |  | -3.9 (0) |  | 0.4 (7) |  | -1.2 (2) |  | **5.7 (92)** |  | -3.9 (0) |  | -0.3 (3) |  | -3.9 (0) |  | 1.9 (15) |  | -3.9 (0) |  |
| Z3370 | prophage CP-933V | [37] |  | 1 (32) | **-2.6 (3)** |  | -2.4 (1) |  | -0.3 (21) |  | -1.7 (9) |  | **5.4 (312)** |  | -1.3 (4) |  | **-2.2 (4)** |  | **-4.4 (1)** |  | 0.7 (30) |  | **-7.8 (0)** |  |
| Z3371 | prophage CP-933V | [37] |  | 1 (6) | **2.0 (16)** |  | -4.2 (0) |  | **2.9 (42)** |  | 1.3 (15) |  | **7.6 (337)** |  | 1.0 (4) |  | -4.2 (0) |  | -0.9 (2) |  | 1.2 (9) |  | -4.2 (0) |  |
| Z3372 | prophage CP-933V |  |  | 1 (14) | 1.4 (22) |  | -5.3 (0) |  | 0.6 (18) |  | 0.0 (13) |  | **5.1 (126)** |  | -5.3 (0) |  | -0.8 (5) |  | 0.9 (15) |  | 0.7 (13) |  | 0.5 (3) |  |
| Z3609 | restricted to *Escherichia* |  |  | 1 (3) | 1.2 (4) |  | -4.7 (0) |  | 1.1 (6) |  | 0.2 (3) |  | **5.4 (35)** |  | -4.7 (0) |  | -4.7 (0) |  | 0.1 (2) |  | **-4.7 (0)** |  | **3.1 (5)** |  |
| Z4174 | hypothetical protein |  |  | 1 (2) | 2.1 (6) |  | -2.1 (0) |  | **4.2 (37)** |  | **3.8 (31)** |  | **5.5 (30)** |  | **3.1 (7)** |  | 1.5 (4) |  | **2.7 (9)** |  | 0.9 (2) |  | -2.1 (0) |  |
| Z4183 | hypothetical protein |  |  | 1 (0) | 0.0 (0) |  | 0.0 (0) |  | **3.4 (12)** |  | 0.0 (0) |  | **5.6 (19)** |  | 0.0 (0) |  | **3.6 (11)** |  | 0.0 (0) |  | 0.0 (0) |  | 0.0 (0) |  |
| Z4201 | hypothetical protein |  |  | 1 (0) | 2.8 (1) |  | 0.0 (0) |  | 0.0 (0) |  | 0.0 (0) |  | **5.6 (3)** |  | 0.0 (0) |  | 0.0 (0) |  | 0.0 (0) |  | 3.0 (1) |  | 0.0 (0) |  |
| Z5018 | Restricted to *Escherichia* and *Salmonella* |  |  | 1 (1) | 1.5 (2) |  | 3.2 (2) |  | 0.4 (1) |  | 1.5 (3) |  | **5.1 (11)** |  | -2.1 (0) |  | 2.4 (4) |  | 2.1 (3) |  | -2.1 (0) |  | -2.1 (0) |  |
| Z5071 | inner membrane protein |  |  | 1 (4) | -0.7 (1) |  | 1.9 (2) |  | 0.5 (5) |  | -0.7 (2) |  | **5.7 (51)** |  | **2.4 (7)** |  | -4.4 (0) |  | 1.2 (5) |  | 0.5 (3) |  | -4.4 (0) |  |
| Z5212 | hypothetical protein |  |  | 1 (0) | 0.0 (0) |  | 0.0 (0) |  | 0.0 (0) |  | 0.0 (0) |  | **5.6 (2)** |  | 0.0 (0) |  | 0.0 (0) |  | 0.0 (0) |  | 0.0 (0) |  | 0.0 (0) |  |
| Z5214 | *espY5‘*, orphan, secreted protein | [39, 115, 93] |  | 1 (2) | -1.5 (0) |  | 2.1 (1) |  | -0.6 (1) |  | -0.6 (1) |  | **6.0 (31)** |  | 1.6 (2) |  | **-5.2 (0)** |  | -1.8 (0) |  | -1.2 (0) |  | **2.7 (2)** |  |
| Z5339 | hypothetical protein |  |  | 1 (3) | 1.3 (4) |  | 2.4 (3) |  | 1.4 (7) |  | 1.7 (9) |  | **5.1 (26)** |  | -2.9 (0) |  | -2.9 (0) |  | 1.9 (6) |  | 1.1 (3) |  | **3.9 (8)** |  |
| Z2783 | hypothetical protein |  | LB-solid | 1 (1) | 0.7 (1) |  | -2.1 (0) |  | 0.4 (1) |  | 0.6 (1) |  | -2.1 (0) |  | **5.2 (8)** |  | -2.1 (0) |  | **2.7 (2)** |  | **3.1 (3)** |  | -2.1 (0) |  |
| Z4570 | hypothetical protein |  |  | 1 (11) | **2.4 (34)** |  | 0.6 (3) |  | **2.7 (61)** |  | 1.4 (26) |  | -0.1 (3) |  | **5.6 (176)** |  | **4.2 (123)** |  | **4.3 (120)** |  | **2.1 (28)** |  | **4.7 (48)** |  |
| Z0359 | hypothetical protein |  | Minimal medium | 1 (0) | -1.4 (0) |  | -1.4 (0) |  | -1.4 (0) |  | -1.4 (0) |  | -1.4 (0) |  | 2.8 (1) |  | **5.4 (13)** |  | -1.4 (0) |  | 1.6 (1) |  | -1.4 (0) |  |
| Z0360 | hypothetical protein |  |  | 1 (2) | 0.1 (2) |  | -3.5 (0) |  | -3.5 (0) |  | 1.6 (7) |  | 2.1 (3) |  | -3.5 (0) |  | **5.2 (60)** |  | -3.5 (0) |  | **2.4 (8)** |  | 2.3 (2) |  |
| Z0726 | hypothetical protein |  |  | 1 (3) | 2.0 (8) |  | -2.6 (0) |  | 2.2 (14) |  | **3.6 (41)** |  | -2.6 (0) |  | 1.6 (3) |  | **7.5 (400)** |  | -2.6 (0) |  | -2.6 (0) |  | **4.8 (17)** |  |
| Z1519 | hypothetical protein |  |  | 1 (10) | **2.1 (26)** |  | **2.6 (10)** |  | 0.8 (15) |  | 1.6 (29) |  | 0.6 (4) |  | -0.3 (3) |  | **5.0 (204)** |  | -0.8 (3) |  | **3.0 (45)** |  | -0.2 (1) |  |
| Z1521 | hypothetical protein |  |  | 1 (11) | **1.7 (24)** |  | **2.4 (10)** |  | 0.2 (12) |  | 1.6 (33) |  | -6.9 (0) |  | 0.2 (5) |  | **5.6 (356)** |  | -0.4 (5) |  | **1.9 (25)** |  | -6.9 (0) |  |
| Z1966 | hypothetical protein |  |  | 1 (1) | 1.0 (1) |  | -2.6 (0) |  | -2.6 (0) |  | 2.3 (3) |  | -2.6 (0) |  | -2.6 (0) |  | **5.8 (26)** |  | -2.6 (0) |  | -2.6 (0) |  | 3.2 (1) |  |
| Z2005 | hypothetical protein |  |  | 1 (0) | **3.7 (2)** |  | 0.0 (0) |  | 2.5 (1) |  | **3.6 (2)** |  | 0.0 (0) |  | 0.0 (0) |  | **5.5 (6)** |  | 3.3 (1) |  | 0.0 (0) |  | 0.0 (0) |  |
| Z2511 | hypothetical protein |  |  | 1 (0) | 0.0 (0) |  | 0.0 (0) |  | 0.0 (0) |  | **3.6 (4)** |  | 0.0 (0) |  | 0.0 (0) |  | **5.1 (7)** |  | 0.0 (0) |  | 3.0 (1) |  | 0.0 (0) |  |
| Z3065 | hypothetical protein |  |  | 1 (19) | 0.1 (13) |  | -6.7 (0) |  | -1.4 (6) |  | **4.0 (297)** |  | 1.5 (15) |  | 1.6 (21) |  | **8.9 (6063)** |  | **2.1 (49)** |  | 0.6 (17) |  | **4.6 (80)** |  |
| Z3066 | hypothetical protein |  |  | 1 (1) | -1.4 (0) |  | -1.4 (0) |  | -1.4 (0) |  | **4.1 (37)** |  | -1.4 (0) |  | -1.4 (0) |  | **7.9 (337)** |  | -1.4 (0) |  | -1.4 (0) |  | -1.4 (0) |  |
| Z4912 | hypothetical protein |  |  | 1 (5) | 1.5 (8) |  | 2.9 (6) |  | 0.9 (7) |  | **3.5 (51)** |  | -3.4 (0) |  | 0.8 (3) |  | **7.4 (532)** |  | 1.4 (7) |  | **2.6 (17)** |  | -3.4 (0) |  |
| Z4915 | hypothetical protein |  |  | 1 (2) | -0.5 (1) |  | -3.2 (0) |  | 0.1 (2) |  | **2.3 (11)** |  | -3.2 (0) |  | 1.0 (2) |  | **6.9 (181)** |  | 1.0 (3) |  | 1.7 (4) |  | -3.2 (0) |  |
| Z4917 | hypothetical protein |  |  | 1 (3) | 1.7 (6) |  | 1.3 (1) |  | -1.5 (1) |  | **2.9 (23)** |  | -4.0 (0) |  | 2.2 (5) |  | **6.7 (207)** |  | -0.7 (1) |  | 0.5 (3) |  | 2.9 (4) |  |
| Z5122 | hypothetical protein |  |  | 1 (175) | -1.7 (35) |  | **-3.5 (3)** |  | **-5.4 (3)** |  | 0.5 (245) |  | **3.9 (743)** |  | -0.3 (52) |  | **5.6 (5816)** |  | -1.3 (45) |  | **-4.8 (4)** |  | -2.0 (8) |  |
| Z2156 | hypothetical protein |  |  | 1 (17) | -1.1 (4) |  | -4.7 (0) |  | **-4.7 (0)** |  | -0.6 (10) |  | 0.9 (8) |  | -4.7 (0) |  | **4.4 (221)** |  | **5.5 (448)** |  | 1.2 (23) |  | -4.7 (0) |  |
| Z3271 | hypothetical protein |  | spinach juice | 1 (9) | -0.5 (4) |  | -4.6 (0) |  | -0.3 (6) |  | 0.7 (14) |  | -4.6 (0) |  | **4.7 (79)** |  | **2.9 (41)** |  | **5.2 (191)** |  | 0.8 (9) |  | **2.8 (10)** |  |
| Z3560 | hypothetical protein |  |  | 1 (1) | 1.7 (2) |  | -3.4 (0) |  | -0.1 (1) |  | 0.7 (1) |  | -3.4 (0) |  | **3.7 (4)** |  | **2.7 (4)** |  | **5.5 (25)** |  | 1.1 (1) |  | -3.4 (0) |  |
| Z4375 | hypothetical protein |  |  | 1 (4) | 1.1 (6) |  | -4.1 (0) |  | -1.6 (1) |  | 1.4 (11) |  | -4.1 (0) |  | 1.7 (5) |  | 1.8 (9) |  | **5.0 (85)** |  | 0.4 (3) |  | 2.8 (5) |  |
| Z4376 | hypothetical protein |  |  | 1 (7) | 0.2 (5) |  | 1.0 (2) |  | 0.7 (9) |  | **2.8 (44)** |  | 1.3 (4) |  | 1.4 (6) |  | **2.2 (18)** |  | **6.4 (320)** |  | **2.0 (15)** |  | 1.5 (3) |  |
| Z4601 | hypothetical protein |  |  | 1 (0) | 2.8 (2) |  | 0.0 (0) |  | 2.5 (3) |  | 0.0 (0) |  | 0.0 (0) |  | **4.2 (4)** |  | **4.6 (9)** |  | **6.6 (36)** |  | **3.9 (5)** |  | 0.0 (0) |  |
| Z4890 | hypothetical protein |  |  | 1 (17) | **2.1 (45)** |  | -0.4 (2) |  | **3.9 (230)** |  | **4.9 (500)** |  | -0.7 (3) |  | -2.1 (1) |  | **4.7 (286)** |  | **5.6 (485)** |  | **2.1 (42)** |  | 0.8 (5) |  |
| Z4909 | hypothetical protein |  |  | 1 (15) | 0.7 (14) |  | -5.9 (0) |  | **2.7 (80)** |  | **2.5 (77)** |  | -5.9 (0) |  | **-5.9 (0)** |  | **2.6 (54)** |  | **5.2 (301)** |  | 0.8 (14) |  | -5.9 (0) |  |
| Z5730 | hypothetical protein |  |  | 1 (4) | -3.3 (0) |  | -3.3 (0) |  | 1.0 (7) |  | 1.9 (14) |  | -3.3 (0) |  | -3.3 (0) |  | 1.3 (6) |  | **5.1 (81)** |  | 1.6 (7) |  | -3.3 (0) |  |
| Z0351 | hypothetical protein |  | radish sprouts | 1 (0) | **4.2 (1)** |  | 0.0 (0) |  | 0.0 (0) |  | **4.6 (3)** |  | 0.0 (0) |  | 0.0 (0) |  | 0.0 (0) |  | 3.3 (1) |  | **5.7 (4)** |  | 0.0 (0) |  |
| Z1023 | *ybiJ*, biofilm formation | [40, 41] |  | 1 (17) | -0.2 (8) |  | 0.2 (3) |  | -2.6 (2) |  | 1.5 (45) |  | **2.5 (25)** |  | -0.9 (3) |  | 1.7 (35) |  | 1.7 (31) |  | **6.6 (945)** |  | **3.3 (28)** |  |
| Z1027 | *ybiM*, biofilm formation | [42] |  | 1 (17) | -0.3 (8) |  | -5.6 (0) |  | **-3.1 (1)** |  | -0.3 (12) |  | 0.0 (4) |  | **-5.6 (0)** |  | **-5.6 (0)** |  | -2.3 (2) |  | **7.5 (1751)** |  | -5.6 (0) |  |
| Z1511 | hypothetical protein |  |  | 1 (0) | 0.0 (0) |  | 0.0 (0) |  | 0.0 (0) |  | 0.0 (0) |  | 0.0 (0) |  | 0.0 (0) |  | 0.0 (0) |  | 0.0 (0) |  | **6.2 (5)** |  | 0.0 (0) |  |
| Z4396 | *ygiD*, biofilm formation | [43] |  | 1 (11) | 0.8 (13) |  | -6.3 (0) |  | 1.7 (33) |  | 1.0 (24) |  | 0.9 (6) |  | **-6.3 (0)** |  | 1.3 (19) |  | 1.0 (14) |  | **5.6 (337)** |  | 1.5 (6) |  |
| Z4455 | hypothetical protein |  |  | 1 (4) | 1.0 (5) |  | -3.9 (0) |  | **4.2 (58)** |  | 2.0 (14) |  | -3.9 (0) |  | -3.9 (0) |  | -3.9 (0) |  | **2.3 (11)** |  | **5.1 (79)** |  | 3.0 (5) |  |
| Z4460 | hypothetical protein |  |  | 1 (5) | -2.1 (1) |  | -4.8 (0) |  | -1.5 (1) |  | -0.3 (4) |  | 1.8 (5) |  | -0.6 (1) |  | -4.8 (0) |  | 0.9 (6) |  | **5.9 (197)** |  | **3.6 (10)** |  |
| Z4807 | *yhhW*, quercetin detoxification | [46] |  | 1 (13) | 0.0 (8) |  | 0.7 (4) |  | **2.1 (47)** |  | 0.4 (17) |  | 1.0 (7) |  | **-6.2 (0)** |  | 0.9 (16) |  | 1.7 (25) |  | **5.8 (430)** |  | -0.3 (2) |  |
| Z5808 | *yjfY*, biofilm formation | [47-49] |  | 1 (13) | **2.7 (50)** |  | 1.5 (6) |  | 0.4 (15) |  | **3.0 (99)** |  | 1.8 (12) |  | **2.1 (19)** |  | -0.3 (7) |  | 1.8 (27) |  | **7.5 (1365)** |  | **4.5 (49)** |  |
| Z0245 | hypothetical protein |  | feces | 1 (0) | **3.7 (2)** |  | 0.0 (0) |  | **3.4 (2)** |  | 0.0 (0) |  | 0.0 (0) |  | 0.0 (0) |  | 0.0 (0) |  | 3.3 (1) |  | 3.0 (1) |  | **5.8 (3)** |  |
| Z0387 | Annot. As „dubious“ |  |  | 1 (3) | 0.1 (2) |  | **4.2 (12)** |  | -2.6 (0) |  | 0.1 (3) |  | 3.0 (8) |  | 2.5 (7) |  | 1.0 (4) |  | -2.6 (0) |  | -2.6 (0) |  | **6.0 (40)** |  |
| Z0706 | hypothetical protein |  |  | 1 (0) | 0.0 (0) |  | 0.0 (0) |  | 0.0 (0) |  | 0.0 (0) |  | 0.0 (0) |  | 0.0 (0) |  | 0.0 (0) |  | 0.0 (0) |  | 0.0 (0) |  | **5.8 (3)** |  |
| Z0742 | Methionine biosynthesis | [44-50] |  | 1 (4) | 0.1 (2) |  | -5.1 (0) |  | 0.5 (5) |  | 0.9 (7) |  | -5.1 (0) |  | -5.1 (0) |  | **4.8 (68)** |  | 1.7 (7) |  | 1.6 (7) |  | **5.1 (23)** |  |
| Z1197 | hypothetical protein |  |  | 1 (0) | 0.0 (0) |  | 0.0 (0) |  | 2.5 (3) |  | 0.0 (0) |  | 0.0 (0) |  | 0.0 (0) |  | **4.6 (9)** |  | 3.3 (3) |  | 0.0 (0) |  | **5.8 (6)** |  |
| Z1517 | hypothetical protein |  |  | 1 (0) | 0.0 (0) |  | 0.0 (0) |  | **3.4 (7)** |  | 0.0 (0) |  | 0.0 (0) |  | 0.0 (0) |  | 0.0 (0) |  | 0.0 (0) |  | 0.0 (0) |  | **5.8 (8)** |  |
| Z1527 | Switch bet. biofilm and planctonic life style, ycdT | [51-53] |  | 1 (7) | -0.6 (3) |  | -6.4 (0) |  | -0.7 (4) |  | 0.5 (10) |  | 1.2 (5) |  | 0.8 (4) |  | **2.5 (28)** |  | 0.9 (8) |  | -1.0 (2) |  | **5.4 (56)** |  |
| Z2119 | hypothetical protein |  |  | 1 (0) | 0.0 (0) |  | 0.0 (0) |  | 2.5 (3) |  | 0.0 (0) |  | 0.0 (0) |  | 0.0 (0) |  | 0.0 (0) |  | 0.0 (0) |  | 0.0 (0) |  | **5.8 (8)** |  |
| Z2199 | hypothetical protein |  |  | 1 (0) | 0.0 (0) |  | 0.0 (0) |  | 0.0 (0) |  | 2.7 (3) |  | 0.0 (0) |  | 0.0 (0) |  | 0.0 (0) |  | 0.0 (0) |  | 0.0 (0) |  | **5.8 (6)** |  |
| Z2368 | Encoded within prophage CP-933R |  |  | 1 (5) | 0.2 (3) |  | -2.6 (0) |  | 0.8 (7) |  | 1.0 (10) |  | -2.6 (0) |  | **4.4 (37)** |  | -2.6 (0) |  | -2.6 (0) |  | 1.9 (11) |  | **6.8 (104)** |  |
| Z2560 | hypothetical protein |  |  | 1 (6) | -0.4 (3) |  | 0.7 (2) |  | 0.2 (6) |  | -0.1 (5) |  | 1.0 (3) |  | 1.9 (7) |  | -1.0 (2) |  | **2.0 (14)** |  | **-4.6 (0)** |  | **5.0 (33)** |  |
| Z2619 | membrane protein, glucuronate metabol. | [38, 56] |  | 1 (0) | -1.4 (0) |  | -1.4 (0) |  | **3.2 (2)** |  | 2.2 (1) |  | -1.4 (0) |  | -1.4 (0) |  | 3.1 (2) |  | -1.4 (0) |  | 2.5 (1) |  | **6.0 (4)** |  |
| Z3722 | Contains functional domain |  |  | 1 (21) | 0.7 (21) |  | 1.8 (12) |  | -1.0 (9) |  | 0.8 (35) |  | **-7.4 (0)** |  | 1.0 (14) |  | 1.3 (33) |  | -1.4 (5) |  | **2.5 (70)** |  | **5.2 (129)** |  |

Table 3 Genes of the LEE pathogenicity island. For each gene, the first number indicates the logFC of a certain condition compared to LB; RPKM values are shown in parentheses. The magnitude of the absolute value of logFC is indicated by shades of grey. Significantly differentially expressed genes are in bold (i.e., p values ≤ 0.05 in *edgeR*).

| gene tag | product | LB | LB-pH9 |  | LB-pH4 |  | LB-15°C |  | LB-nitrite |  | LB-antibiotics |  | LB-solid |  | minimal medium |  | spinach juice |  | radish sprouts |  | faeces |  |
| --- | --- | --- | --- | --- | --- | --- | --- | --- | --- | --- | --- | --- | --- | --- | --- | --- | --- | --- | --- | --- | --- | --- |
| Z5100 | hypothetical protein | 1 (20) | **-6.8 (0)** |  | -1.5 (1) |  | **-6.8 (0)** |  | **-3.2 (2)** |  | 1.8 (17) |  | **-6.8 (0)** |  | -1.0 (6) |  | **-3.5 (1)** |  | **-6.8 (0)** |  | -6.8 (0) |  |
| Z5102 | hypothetical protein | 1 (149) | **-5.5 (2)** |  | -0.7 (15) |  | **-4.9 (4)** |  | **-4.1 (7)** |  | 0.1 (41) |  | **-8.3 (0)** |  | -0.2 (81) |  | **-4.1 (5)** |  | **-3.4 (8)** |  | **-8.3 (0)** |  |
| Z5103 | hypothetical protein | 1 (128) | **-3.0 (10)** |  | **-7.9 (0)** |  | **-4.0 (7)** |  | **-3.0 (15)** |  | 1.7 (117) |  | **-7.9 (0)** |  | 1.1 (187) |  | **-3.6 (6)** |  | **-4.9 (2)** |  | **-7.9 (0)** |  |
| Z5104 | hypothetical protein | 1 (463) | **-3.9 (17)** |  | **-3.6 (6)** |  | **-2.6 (63)** |  | **-2.2 (91)** |  | -0.5 (80) |  | **-3.7 (11)** |  | 0.6 (431) |  | **-4.3 (13)** |  | **-3.3 (26)** |  | **-4.6 (3)** |  |
| Z5105 | secreted protein EspB | 1 (903) | **-3.5 (46)** |  | **-4.6 (6)** |  | **-4.5 (35)** |  | -1.5 (302) |  | **-2.0 (57)** |  | **-3.8 (21)** |  | 0.9 (1070) |  | **-4.3 (26)** |  | **-3.1 (55)** |  | **-6.8 (1)** |  |
| Z5106 | secreted protein EspD | 1 (847) | **-4.8 (19)** |  | **-5.0 (4)** |  | **-6.2 (10)** |  | **-2.8 (119)** |  | -0.5 (155) |  | **-4.3 (15)** |  | 1.0 (1070) |  | **-4.4 (24)** |  | **-3.8 (32)** |  | **-12.9 (0)** |  |
| Z5107 | secreted protein EspA | 1 (1035) | **-4.5 (31)** |  | **-7.0 (1)** |  | **-7.7 (4)** |  | **-2.7 (161)** |  | 1.6 (888) |  | **-3.1 (45)** |  | 1.4 (1819) |  | **-4.1 (37)** |  | **-3.4 (58)** |  | **-12.3 (0)** |  |
| Z5108 | hypothetical protein | 1 (324) | **-4.8 (7)** |  | **-5.1 (2)** |  | **-11.4 (0)** |  | **-3.2 (34)** |  | 0.2 (97) |  | **-3.4 (11)** |  | 0.8 (347) |  | **-4.5 (8)** |  | **-4.0 (12)** |  | **-3.0 (7)** |  |
| Z5109 | hypothetical protein | 1 (32) | **-2.6 (3)** |  | **-8.2 (0)** |  | **-5.7 (0)** |  | **-2.9 (4)** |  | -1.0 (4) |  | -0.6 (7) |  | 1.4 (52) |  | **-3.0 (2)** |  | **-3.3 (2)** |  | -2.3 (1) |  |
| Z5110 | intimin adherence protein | 1 (392) | **-4.7 (9)** |  | **-4.5 (3)** |  | **-5.7 (6)** |  | **-3.1 (45)** |  | 1.2 (244) |  | **-3.9 (9)** |  | 0.6 (376) |  | **-4.6 (9)** |  | **-3.4 (20)** |  | **-7.3 (0)** |  |
| Z5111 | hypothetical protein | 1 (363) | **-5.8 (4)** |  | **-5.1 (2)** |  | **-5.6 (7)** |  | **-3.5 (31)** |  | **2.6 (608)** |  | **-4.7 (5)** |  | 1.1 (523) |  | **-4.9 (7)** |  | **-3.9 (13)** |  | **-10.4 (0)** |  |
| Z5112 | putative translocated intimin receptor protein | 1 (247) | **-3.4 (15)** |  | **-4.4 (2)** |  | **-4.8 (8)** |  | -1.8 (67) |  | 1.2 (149) |  | **-2.9 (11)** |  | **1.9 (594)** |  | **-3.5 (12)** |  | **-3.2 (15)** |  | **-11.7 (0)** |  |
| Z5113 | hypothetical protein | 1 (337) | **-3.0 (25)** |  | -2.0 (15) |  | **-2.9 (40)** |  | -1.7 (103) |  | 1.1 (194) |  | **-2.3 (23)** |  | **1.7 (689)** |  | **-2.5 (36)** |  | **-4.2 (10)** |  | **-3.3 (6)** |  |
| Z5114 | hypothetical protein | 1 (55) | **-2.3 (7)** |  | -0.2 (9) |  | **-2.9 (7)** |  | -1.8 (16) |  | **2.7 (102)** |  | -1.3 (8) |  | **2.3 (183)** |  | -2.0 (9) |  | **-3.5 (3)** |  | **-7.5 (0)** |  |
| Z5115 | hypothetical protein | 1 (111) | **-2.9 (10)** |  | -2.0 (5) |  | **-3.6 (8)** |  | **-1.9 (30)** |  | 1.5 (90) |  | -1.3 (16) |  | **1.6 (231)** |  | **-2.7 (11)** |  | **-2.8 (9)** |  | -0.1 (19) |  |
| Z5116 | hypothetical protein | 1 (60) | **-2.6 (6)** |  | **-2.6 (2)** |  | **-5.5 (1)** |  | **-2.9 (8)** |  | 1.4 (44) |  | -0.4 (16) |  | 0.8 (71) |  | **-4.1 (2)** |  | **-2.1 (9)** |  | 0.3 (13) |  |
| Z5117 | hypothetical protein | 1 (53) | **-4.1 (2)** |  | **-6.9 (0)** |  | **-4.4 (2)** |  | **-2.7 (7)** |  | 1.1 (29) |  | -2.7 (3) |  | 0.6 (52) |  | **-2.6 (5)** |  | **-6.9 (0)** |  | -6.9 (0) |  |
| Z5118 | hypothetical protein | 1 (69) | **-4.9 (1)** |  | -2.3 (2) |  | **-7.6 (0)** |  | **-4.0 (4)** |  | 1.1 (39) |  | **-7.6 (0)** |  | -0.1 (38) |  | **-2.8 (5)** |  | **-2.4 (7)** |  | **-7.6 (0)** |  |
| Z5119 | hypothetical protein | 1 (79) | **-2.2 (10)** |  | **-3.4 (1)** |  | **-3.4 (6)** |  | **-2.0 (19)** |  | 0.5 (29) |  | **-2.2 (6)** |  | 0.8 (84) |  | **-3.7 (4)** |  | **-3.7 (4)** |  | -0.9 (7) |  |
| Z5120 | hypothetical protein | 1 (37) | **-3.1 (3)** |  | **-3.0 (1)** |  | **-3.5 (3)** |  | **-2.3 (7)** |  | 0.7 (17) |  | -1.5 (4) |  | **1.8 (83)** |  | **-2.7 (3)** |  | **-3.8 (2)** |  | **-9.3 (0)** |  |
| Z5121 | hypothetical protein | 1 (29) | **-3.8 (1)** |  | -6.5 (0) |  | **-4.0 (1)** |  | -2.0 (8) |  | **2.1 (37)** |  | -1.3 (4) |  | **1.9 (79)** |  | -1.3 (8) |  | **-6.5 (0)** |  | 0.9 (10) |  |
| Z5122 | hypothetical protein | 1 (175) | -1.7 (35) |  | **-3.5 (3)** |  | **-5.4 (3)** |  | 0.5 (245) |  | **3.9 (743)** |  | -0.3 (52) |  | **5.6 (5816)** |  | -1.3 (45) |  | **-4.8 (4)** |  | -2.0 (8) |  |
| Z5123 | hypothetical protein | 1 (41) | **-3.4 (2)** |  | -1.8 (2) |  | **-7.1 (0)** |  | -1.7 (11) |  | **2.8 (72)** |  | -1.3 (5) |  | **2.7 (166)** |  | -1.9 (6) |  | **-7.1 (0)** |  | -7.1 (0) |  |
| Z5124 | hypothetical protein | 1 (37) | **-3.8 (2)** |  | **-7.4 (0)** |  | **-4.9 (1)** |  | -1.4 (13) |  | **3.0 (80)** |  | -2.2 (3) |  | **2.2 (106)** |  | -1.2 (9) |  | **-2.5 (4)** |  | **-7.4 (0)** |  |
| Z5125 | hypothetical protein | 1 (80) | **-4.0 (3)** |  | **-8.2 (0)** |  | **-4.3 (3)** |  | -1.4 (30) |  | **1.8 (75)** |  | **-4.0 (2)** |  | **1.8 (186)** |  | -2.0 (12) |  | **-4.3 (2)** |  | -2.4 (3) |  |
| Z5126 | hypothetical protein | 1 (50) | **-4.4 (1)** |  | **-4.0 (1)** |  | **-6.8 (0)** |  | **-2.4 (9)** |  | **2.5 (78)** |  | -1.2 (8) |  | **1.7 (104)** |  | **-2.8 (4)** |  | **-4.1 (2)** |  | **-9.3 (0)** |  |
| Z5127 | hypothetical protein | 1 (91) | **-4.3 (3)** |  | **-8.5 (0)** |  | **-8.5 (0)** |  | **-2.6 (15)** |  | 1.7 (82) |  | **-2.7 (5)** |  | 1.2 (143) |  | **-2.5 (10)** |  | **-3.6 (5)** |  | **-8.5 (0)** |  |
| Z5128 | hypothetical protein | 1 (74) | **-2.5 (7)** |  | **-7.9 (0)** |  | **-5.4 (1)** |  | **-5.2 (2)** |  | **3.1 (165)** |  | -2.2 (5) |  | -0.1 (44) |  | -1.6 (15) |  | **-2.7 (6)** |  | **-7.9 (0)** |  |
| Z5129 | negative regulator GrlR | 1 (118) | **-2.4 (14)** |  | **-8.5 (0)** |  | **-5.1 (3)** |  | **-3.9 (7)** |  | **3.7 (429)** |  | -0.5 (30) |  | 0.9 (148) |  | -1.0 (36) |  | **-4.6 (3)** |  | -2.7 (3) |  |
| Z5131 | hypothetical protein | 1 (12) | **-5.5 (0)** |  | -5.5 (0) |  | -2.1 (2) |  | **-2.8 (1)** |  | **3.4 (35)** |  | -0.3 (3) |  | 1.0 (16) |  | -1.2 (3) |  | **-5.5 (0)** |  | 1.3 (5) |  |
| Z5132 | secretion system apparatus protein SsaU | 1 (41) | **-5.5 (0)** |  | **-8.3 (0)** |  | **-8.3 (0)** |  | **-4.7 (1)** |  | 0.1 (11) |  | **-8.3 (0)** |  | **-2.5 (4)** |  | **-5.0 (1)** |  | **-4.3 (1)** |  | **-8.3 (0)** |  |
| Z5133 | hypothetical protein | 1 (47) | **-4.5 (1)** |  | **-8.2 (0)** |  | **-5.7 (1)** |  | **-5.5 (1)** |  | 0.2 (15) |  | -1.7 (5) |  | -1.9 (8) |  | **-2.7 (4)** |  | **-5.2 (1)** |  | **-8.2 (0)** |  |
| Z5134 | hypothetical protein | 1 (62) | **-7.1 (0)** |  | **-7.1 (0)** |  | **-7.1 (0)** |  | **-4.4 (3)** |  | -1.5 (6) |  | **-2.9 (3)** |  | -1.6 (13) |  | **-3.8 (2)** |  | **-7.1 (0)** |  | -7.1 (0) |  |
| Z5135 | type III secretion system protein | 1 (95) | **-6.2 (1)** |  | **-8.9 (0)** |  | **-8.9 (0)** |  | **-3.6 (7)** |  | 0.6 (37) |  | **-2.2 (7)** |  | -1.0 (29) |  | **-4.7 (2)** |  | **-5.0 (2)** |  | **-8.9 (0)** |  |
| Z5136 | hypothetical protein | 1 (149) | **-9.5 (0)** |  | **-9.5 (0)** |  | **-6.1 (2)** |  | **-4.3 (7)** |  | -0.3 (32) |  | **-4.3 (2)** |  | -0.7 (56) |  | **-5.2 (2)** |  | **-4.0 (5)** |  | **-9.5 (0)** |  |
| Z5137 | hypothetical protein | 1 (232) | **-5.2 (4)** |  | **-3.2 (4)** |  | **-4.8 (7)** |  | **-4.0 (14)** |  | 0.5 (84) |  | **-2.8 (11)** |  | -0.8 (84) |  | **-4.1 (8)** |  | **-3.1 (15)** |  | **-4.3 (2)** |  |
| Z5138 | hypothetical protein | 1 (438) | **-5.4 (5)** |  | **-3.7 (5)** |  | **-10.0 (0)** |  | **-4.5 (17)** |  | -0.8 (60) |  | **-3.5 (12)** |  | -1.6 (84) |  | **-5.2 (6)** |  | **-4.1 (13)** |  | **-3.2 (8)** |  |
| Z5139 | hypothetical protein | 1 (899) | **-6.8 (4)** |  | **-10.4 (0)** |  | **-10.4 (0)** |  | **-5.6 (16)** |  | -1.2 (89) |  | **-2.8 (37)** |  | **-2.6 (82)** |  | **-5.6 (9)** |  | **-5.2 (12)** |  | **-3.6 (11)** |  |
| Z5140 | hypothetical protein | 1 (597) | **-4.0 (23)** |  | **-3.2 (11)** |  | **-10.9 (0)** |  | **-3.1 (67)** |  | 0.6 (246) |  | -1.8 (59) |  | -0.9 (208) |  | **-4.3 (17)** |  | **-4.0 (20)** |  | **-3.4 (9)** |  |
| Z5142 | hypothetical protein | 1 (51) | -1.8 (9) |  | -2.0 (2) |  | **-2.8 (6)** |  | -1.0 (23) |  | -0.1 (12) |  | -1.6 (6) |  | 1.2 (75) |  | **-2.5 (5)** |  | **-2.3 (6)** |  | -1.5 (3) |  |
| Z5143 | hypothetical protein | 1 (6) | **-5.2 (0)** |  | -5.2 (0) |  | **-5.2 (0)** |  | **-5.2 (0)** |  | -5.2 (0) |  | -5.2 (0) |  | 1.6 (11) |  | **-5.2 (0)** |  | **-5.2 (0)** |  | -5.2 (0) |  |

Table 4 Genes compared to LB with high logFCs in feces (see chapter “persistence in cow dung”). For each gene, the first number indicates the logFC of a certain condition compared to LB; RPKM values are shown in parentheses. The magnitude of the absolute value of logFC is indicated by shades of grey. Significantly differentially expressed genes are in bold (i.e., p values ≤ 0.05 in *edgeR*).

| gene tag | product | LB | LB-pH9 |  | LB-pH4 |  | LB-15°C |  | LB-nitrite |  | LB-antibiotics |  | LB-solid |  | minimal medium |  | spinach juice |  | radish sprouts |  | faeces |  |
| --- | --- | --- | --- | --- | --- | --- | --- | --- | --- | --- | --- | --- | --- | --- | --- | --- | --- | --- | --- | --- | --- | --- |
| Z0014 | molecular chaperone DnaK | 1 (185) | -1.0 (55) |  | 0.1 (33) |  | 1.6 (470) |  | 1.6 (520) |  | 0.5 (70) |  | 0.8 (109) |  | 0.0 (120) |  | **3.6 (1290)** |  | 1.2 (248) |  | **3.7 (398)** |  |
| Z0015 | chaperone protein DnaJ | 1 (74) | -1.4 (16) |  | -1.4 (4) |  | 1.3 (150) |  | 0.8 (120) |  | -0.9 (10) |  | 0.0 (23) |  | -0.5 (31) |  | 0.9 (78) |  | 1.2 (99) |  | **4.2 (220)** |  |
| Z0045 | crotonobetainyl-CoA dehydrogenase | 1 (7) | 1.3 (10) |  | 0.4 (1) |  | -0.1 (5) |  | 0.9 (12) |  | 0.7 (3) |  | **2.7 (14)** |  | 1.2 (9) |  | **2.5 (23)** |  | -1.5 (1) |  | **5.8 (63)** |  |
| Z2477 | thiosulfate:cyanide sulfurtransferase | 1 (558) | 0.4 (470) |  | -0.1 (94) |  | 0.9 (963) |  | 0.1 (603) |  | **-2.3 (31)** |  | -0.7 (121) |  | **-2.2 (83)** |  | -1.6 (113) |  | -0.4 (252) |  | **2.4 (537)** |  |
| Z2478 | peripheral inner membrane phage-shock protein | 1 (17) | 1.4 (28) |  | -4.9 (0) |  | **2.0 (60)** |  | 1.2 (37) |  | -4.9 (0) |  | 1.6 (17) |  | 0.6 (16) |  | 0.6 (15) |  | **4.3 (198)** |  | **7.7 (598)** |  |
| Z2479 | DNA-binding transcriptional activator PspC | 1 (17) | 1.5 (31) |  | -0.4 (2) |  | **1.9 (57)** |  | 1.4 (46) |  | -0.1 (5) |  | **-5.7 (0)** |  | 0.8 (20) |  | -0.9 (6) |  | **4.1 (182)** |  | **7.2 (470)** |  |
| Z2480 | phage shock protein B | 1 (25) | 1.4 (39) |  | -0.2 (4) |  | **1.9 (80)** |  | 1.2 (55) |  | -5.5 (0) |  | -0.3 (7) |  | -0.9 (8) |  | 0.0 (15) |  | **3.9 (228)** |  | **6.8 (482)** |  |
| Z2482 | phage shock protein PspA | 1 (64) | 0.8 (63) |  | 0.8 (18) |  | **2.0 (206)** |  | 0.5 (81) |  | -0.4 (12) |  | 0.6 (31) |  | 0.6 (59) |  | 0.6 (56) |  | **4.8 (1077)** |  | **6.5 (970)** |  |
| Z2611 | DNA replication terminus site-binding protein Tus | 1 (9) | -0.4 (4) |  | 1.3 (4) |  | **1.8 (26)** |  | 0.8 (15) |  | -6.0 (0) |  | 0.2 (3) |  | -1.4 (2) |  | -1.2 (2) |  | 0.7 (9) |  | **5.0 (47)** |  |
| Z2876 | heat shock protein HtpX | 1 (93) | 1.4 (154) |  | 0.2 (19) |  | **1.8 (284)** |  | 1.0 (187) |  | -0.2 (22) |  | **3.0 (262)** |  | 0.0 (60) |  | -0.3 (46) |  | 0.1 (59) |  | **3.9 (246)** |  |
| Z2900 | DNA damage-inducible protein YebG | 1 (112) | -0.1 (61) |  | 1.3 (42) |  | -0.1 (83) |  | 0.3 (123) |  | **2.8 (196)** |  | 0.8 (59) |  | 0.6 (99) |  | 0.0 (60) |  | 0.8 (113) |  | **3.7 (226)** |  |
| Z3886 | protein disaggregation chaperone ClpB | 1 (239) | -1.6 (44) |  | **2.4 (194)** |  | 0.5 (267) |  | 0.3 (265) |  | -0.1 (56) |  | 0.9 (140) |  | -0.4 (106) |  | **2.7 (827)** |  | 1.1 (292) |  | **2.5 (216)** |  |
| Z4227 | isopentenyl-diphosphate delta-isomerase | 1 (22) | **1.9 (52)** |  | 0.3 (5) |  | 1.2 (43) |  | **2.0 (89)** |  | -6.6 (0) |  | 1.2 (18) |  | 0.2 (16) |  | 1.6 (40) |  | **1.5 (38)** |  | **5.6 (180)** |  |
| Z4291 | 16S ribosomal RNA methyltransferase RsmE | 1 (57) | -1.0 (17) |  | 1.0 (18) |  | -0.9 (26) |  | -0.8 (30) |  | 0.9 (27) |  | -1.4 (7) |  | -0.1 (33) |  | 1.1 (70) |  | 0.0 (34) |  | **4.1 (165)** |  |
| Z4401 | glycogen synthesis protein GlgS | 1 (240) | 0.4 (182) |  | -0.2 (32) |  | -0.4 (141) |  | 1.2 (480) |  | -0.3 (47) |  | **4.0 (1226)** |  | 0.5 (196) |  | -0.3 (104) |  | **2.6 (838)** |  | **6.6 (3617)** |  |
| Z5182 | heat shock chaperone IbpB | 1 (26) | -0.2 (13) |  | -0.1 (4) |  | 0.4 (29) |  | 0.3 (29) |  | 1.5 (19) |  | **2.5 (47)** |  | -0.3 (13) |  | 1.0 (30) |  | **2.4 (80)** |  | **5.7 (224)** |  |
| Z5183 | heat shock protein IbpA | 1 (26) | 0.5 (22) |  | 1.7 (14) |  | **2.1 (96)** |  | 1.2 (55) |  | 1.5 (20) |  | **2.6 (54)** |  | 0.1 (17) |  | **3.0 (125)** |  | **2.2 (72)** |  | **6.9 (509)** |  |
| Z5458 | periplasmic repressor CpxP | 1 (25) | **3.2 (127)** |  | 1.7 (13) |  | 0.0 (20) |  | 1.1 (47) |  | **2.9 (45)** |  | **4.6 (191)** |  | -0.2 (13) |  | -2.3 (3) |  | **2.5 (84)** |  | **6.0 (260)** |  |

**Table 5 Genes compared to LB either with highest logFC or RPKM values on radish sprouts and in spinach medium or genes known from an association to plants.** For each gene, the first number indicates the logFC of a certain condition compared to LB; RPKM values are shown in parentheses. The magnitude of the absolute value of logFC is indicated by shades of grey. Significantly differentially expressed genes are in bold (i.e., p values ≤ 0.05 in *edgeR*). Note that Z3511-Z3515 are not only active on sprouts, but also in LB medium.

| gene tag | product | LB | LB-pH9 |  | LB-pH4 |  | LB-15°C |  | LB-nitrite |  | LB-antibiotics |  | LB-solid |  | minimal medium |  | spinach juice |  | radish sprouts |  | faeces |  |
| --- | --- | --- | --- | --- | --- | --- | --- | --- | --- | --- | --- | --- | --- | --- | --- | --- | --- | --- | --- | --- | --- | --- |
| Z0070 | L-arabinose isomerase | 1 (4) | -0.5 (2) |  | 1.2 (2) |  | -1.3 (1) |  | 1.7 (13) |  | 0.0 (1) |  | **-5.6 (0)** |  | -1.1 (1) |  | 0.1 (3) |  | **3.1 (23)** |  | -5.6 (0) |  |
| Z1106 | HCP oxidoreductase, NADH-dependent | 1 (3) | -1.1 (1) |  | -4.8 (0) |  | **-4.8 (0)** |  | 0.3 (4) |  | -4.8 (0) |  | 0.4 (2) |  | 0.7 (4) |  | **7.7 (443)** |  | **2.1 (9)** |  | 1.1 (1) |  |
| Z1107 | hydroxylamine reductase | 1 (2) | 0.2 (1) |  | -4.7 (0) |  | **2.1 (7)** |  | 0.2 (2) |  | -4.7 (0) |  | 1.5 (2) |  | -1.1 (1) |  | **8.3 (356)** |  | **2.3 (5)** |  | -4.7 (0) |  |
| Z1109 | aquaporin Z | 1 (9) | **3.0 (41)** |  | 1.3 (4) |  | **4.1 (130)** |  | **3.4 (88)** |  | -5.6 (0) |  | -1.4 (1) |  | 1.1 (12) |  | 0.2 (6) |  | **3.8 (70)** |  | -5.6 (0) |  |
| Z1390 | hydrogenase 1 large subunit | 1 (2) | 0.2 (1) |  | -5.0 (0) |  | -1.1 (1) |  | 1.0 (4) |  | -5.0 (0) |  | -5.0 (0) |  | 1.3 (4) |  | **7.6 (255)** |  | 0.5 (2) |  | -5.0 (0) |  |
| Z1670 | curli production assembly/transport component, 2nd curli operon | 1 (24) | 1.6 (43) |  | 1.0 (8) |  | **2.1 (88)** |  | 1.2 (54) |  | **-7.3 (0)** |  | **-3.1 (1)** |  | 0.3 (18) |  | -0.9 (7) |  | 1.2 (32) |  | 0.5 (6) |  |
| Z1671 | curli assembly protein CsgF | 1 (7) | **2.3 (21)** |  | -4.6 (0) |  | **2.0 (26)** |  | -0.4 (5) |  | -4.6 (0) |  | -4.6 (0) |  | 0.9 (9) |  | -4.6 (0) |  | **2.4 (24)** |  | -4.6 (0) |  |
| Z1672 | curli assembly protein CsgE | 1 (12) | **2.2 (35)** |  | **2.3 (11)** |  | 1.5 (30) |  | 0.9 (23) |  | -5.3 (0) |  | -0.1 (4) |  | 1.2 (19) |  | -2.0 (2) |  | **2.2 (34)** |  | -5.3 (0) |  |
| Z1673 | DNA-binding transcriptional regulator CsgD | 1 (39) | **1.9 (83)** |  | 1.6 (19) |  | 0.0 (33) |  | 0.4 (50) |  | **-7.6 (0)** |  | **-3.4 (1)** |  | 0.6 (37) |  | **-3.4 (2)** |  | 1.3 (56) |  | -1.8 (2) |  |
| Z1675 | curlin minor subunit | 1 (20) | **3.1 (114)** |  | -6.3 (0) |  | **2.9 (138)** |  | **2.9 (156)** |  | 0.4 (7) |  | -1.1 (3) |  | **2.5 (75)** |  | 1.2 (28) |  | **6.0 (803)** |  | -0.4 (3) |  |
| Z1676 | cryptic curlin major subunit | 1 (46) | **5.3 (1143)** |  | 0.6 (13) |  | **2.0 (168)** |  | **3.3 (472)** |  | 0.5 (18) |  | -0.5 (11) |  | **3.8 (440)** |  | -0.3 (23) |  | **5.6 (1336)** |  | **-7.5 (0)** |  |
| Z1697 | biofilm formation regulatory protein BssS | 1 (113) | **2.4 (386)** |  | **2.5 (116)** |  | **6.7 (10874)** |  | **4.4 (2326)** |  | 1.5 (89) |  | **3.7 (512)** |  | 1.5 (219) |  | **3.0 (556)** |  | **6.1 (4555)** |  | **4.7 (530)** |  |
| Z2243 | nitrite extrusion protein 2 | 1 (0) | 2.5 (1) |  | 3.2 (1) |  | **5.8 (19)** |  | **4.4 (8)** |  | -2.1 (0) |  | -2.1 (0) |  | 2.4 (1) |  | 2.1 (1) |  | **7.0 (30)** |  | 3.7 (1) |  |
| Z2315 | azoreductase | 1 (21) | -1.1 (6) |  | -0.4 (3) |  | 0.5 (25) |  | -0.8 (11) |  | 1.8 (19) |  | 0.8 (12) |  | 1.5 (39) |  | 1.1 (25) |  | **4.1 (213)** |  | **2.9 (26)** |  |
| Z2479 | DNA-binding transcriptional activator PspC | 1 (17) | 1.5 (31) |  | -0.4 (2) |  | **1.9 (57)** |  | 1.4 (46) |  | -0.1 (5) |  | **-5.7 (0)** |  | 0.8 (20) |  | -0.9 (6) |  | **4.1 (182)** |  | **7.2 (470)** |  |
| Z2480 | phage shock protein B | 1 (25) | 1.4 (39) |  | -0.2 (4) |  | **1.9 (80)** |  | 1.2 (55) |  | -5.5 (0) |  | -0.3 (7) |  | -0.9 (8) |  | 0.0 (15) |  | **3.9 (228)** |  | **6.8 (482)** |  |
| Z2482 | phage shock protein PspA | 1 (64) | 0.8 (63) |  | 0.8 (18) |  | **2.0 (206)** |  | 0.5 (81) |  | -0.4 (12) |  | 0.6 (31) |  | 0.6 (59) |  | 0.6 (56) |  | **4.8 (1077)** |  | **6.5 (970)** |  |
| Z2591 | acid shock protein precursor | 1 (37) | -0.9 (13) |  | **5.3 (261)** |  | **-6.6 (0)** |  | -0.9 (20) |  | -6.6 (0) |  | **-6.6 (0)** |  | -1.5 (9) |  | **1.8 (80)** |  | **7.5 (4178)** |  | -6.6 (0) |  |
| Z2949 | trehalose-6-phosphate synthase | 1 (20) | **2.0 (49)** |  | 0.3 (4) |  | 0.9 (32) |  | **3.0 (151)** |  | -2.2 (1) |  | 0.1 (7) |  | 1.0 (26) |  | **2.3 (57)** |  | **2.6 (74)** |  | 1.3 (9) |  |
| Z2950 | trehalose-6-phosphate phosphatase | 1 (16) | **2.7 (66)** |  | 0.6 (4) |  | 1.5 (41) |  | **3.2 (141)** |  | -1.1 (2) |  | -0.2 (5) |  | 1.0 (20) |  | **2.2 (45)** |  | **3.7 (133)** |  | 1.4 (8) |  |
| Z2951 | partial high-affinity L-arabinose transport system; membrane protein, fragment 2 | 1 (4) | 0.0 (3) |  | -4.6 (0) |  | -0.6 (2) |  | 0.6 (6) |  | -4.6 (0) |  | -4.6 (0) |  | -4.6 (0) |  | -4.6 (0) |  | **2.3 (12)** |  | -4.6 (0) |  |
| Z2953 | L-arabinose transporter ATP-binding protein | 1 (8) | 1.4 (13) |  | 0.7 (2) |  | 0.4 (9) |  | **1.7 (27)** |  | -6.6 (0) |  | -0.5 (2) |  | -1.1 (2) |  | -0.4 (4) |  | **1.5 (14)** |  | -6.6 (0) |  |
| Z2954 | L-arabinose-binding periplasmic protein | 1 (43) | 1.4 (70) |  | -2.1 (2) |  | -0.3 (29) |  | 1.2 (95) |  | **-8.4 (0)** |  | **-2.6 (2)** |  | **-4.8 (1)** |  | **-3.2 (3)** |  | 1.3 (60) |  | **-8.4 (0)** |  |
| Z3425 | PTS system fructose-specific transporter subunits IIBC | 1 (3) | **1.8 (7)** |  | 1.5 (1) |  | 1.5 (8) |  | 1.3 (7) |  | -5.4 (0) |  | -0.2 (1) |  | 0.4 (3) |  | **5.9 (112)** |  | **3.2 (17)** |  | -5.4 (0) |  |
| Z3426 | 1-phosphofructokinase | 1 (4) | 0.0 (2) |  | -4.9 (0) |  | **2.1 (15)** |  | 1.2 (9) |  | -4.9 (0) |  | 0.3 (2) |  | 1.6 (8) |  | **5.9 (139)** |  | **2.7 (16)** |  | -4.9 (0) |  |
| Z3427 | bifunctional PTS system fructose-specific transporter subunit IIA/HPr protein | 1 (2) | 0.9 (2) |  | -4.3 (0) |  | 1.2 (4) |  | -0.6 (1) |  | -4.3 (0) |  | **2.7 (5)** |  | 1.3 (3) |  | **7.3 (192)** |  | **4.0 (21)** |  | -4.3 (0) |  |
| Z3511 | UDP-4-amino-4-deoxy-L-arabinose--oxoglutarate aminotransferase | 1 (122) | **-1.9 (19)** |  | **-2.2 (4)** |  | -0.4 (76) |  | -1.0 (58) |  | -0.8 (17) |  | **-2.9 (5)** |  | **-4.0 (5)** |  | **-4.8 (2)** |  | 0.3 (87) |  | **-4.2 (1)** |  |
| Z3512 | undecaprenyl phosphate 4-deoxy-4-formamido-L-arabinose transferase | 1 (75) | **-2.5 (7)** |  | -0.5 (9) |  | -1.8 (18) |  | **-2.2 (15)** |  | -1.5 (7) |  | **-3.9 (2)** |  | **-5.5 (1)** |  | **-3.6 (3)** |  | -0.7 (27) |  | **-3.2 (1)** |  |
| Z3513 | bifunctional UDP-glucuronic acid decarboxylase/UDP-4-amino-4-deoxy-L-arabinose formyltransferase | 1 (88) | **-3.3 (5)** |  | -1.7 (4) |  | -1.3 (29) |  | **-2.2 (17)** |  | **-2.4 (4)** |  | **-3.2 (3)** |  | **-2.8 (7)** |  | **-4.3 (2)** |  | -0.4 (39) |  | **-4.5 (1)** |  |
| Z3515 | 4-amino-4-deoxy-L-arabinose transferase | 1 (64) | -1.8 (11) |  | -1.6 (3) |  | 0.7 (91) |  | -0.7 (39) |  | -1.8 (5) |  | -1.8 (6) |  | **-2.4 (8)** |  | **-3.0 (5)** |  | -0.4 (28) |  | **-9.7 (0)** |  |
| Z5648 | phage shock protein G | 1 (2) | **3.1 (11)** |  | **6.4 (31)** |  | **4.0 (29)** |  | **2.8 (14)** |  | **4.5 (13)** |  | 2.1 (3) |  | **3.0 (11)** |  | 2.2 (6) |  | **7.7 (257)** |  | **8.5 (140)** |  |
| Z5717 | arginine:agmatin antiporter | 1 (5) | 0.2 (3) |  | -5.7 (0) |  | **-3.2 (0)** |  | 1.3 (11) |  | -5.7 (0) |  | -0.5 (1) |  | **3.9 (46)** |  | **7.5 (528)** |  | **1.8 (9)** |  | -5.7 (0) |  |
| Z5719 | biodegradative arginine decarboxylase | 1 (3) | -0.1 (2) |  | 0.3 (1) |  | -1.7 (1) |  | -1.4 (1) |  | -6.0 (0) |  | **-6.0 (0)** |  | 0.3 (3) |  | **8.3 (647)** |  | 1.2 (4) |  | -6.0 (0) |  |
| Z5734 | lysine decarboxylase 1 | 1 (1) | 1.6 (2) |  | -4.0 (0) |  | -0.1 (1) |  | 0.6 (1) |  | -4.0 (0) |  | -4.0 (0) |  | -4.0 (0) |  | **7.6 (107)** |  | 0.5 (1) |  | -4.0 (0) |  |
| Z5735 | lysine/cadaverine antiporter | 1 (0) | 0.6 (0) |  | 3.2 (1) |  | -2.1 (0) |  | 0.6 (1) |  | -2.1 (0) |  | 2.1 (1) |  | -2.1 (0) |  | **5.3 (10)** |  | -2.1 (0) |  | -2.1 (0) |  |
