# Supplementary material for: Comparison of strand-specific transcriptomes of enterohemorrhagic Escherichia coli O157:H7 EDL933 (EHEC) under eleven different environmental conditions including radish sprouts and cattle feces
Source: BMC Genomics. 2014 May 9;15:353. doi: 10.1186/1471-2164-15-353 (PMC4048457; doi:10.1186/1471-2164-15-353)
Supplement: Supplementary file 5 — Additional file 5: Table S5: Transcriptional regulation of the virulence associated genes. (DOCX 32 KB) [file 12864_2013_6096_MOESM5_ESM.docx]

**Table S5. Virulence associated genes.** For each gene, the first number indicates the logFC of a certain condition compared to LB; RPKM values are shown in parentheses. The magnitude of the absolute value of logFC is indicated by shades of grey. Significantly differentially expressed genes are in bold (i.e., p values ≤ 0.05 in *edgeR*). Genes below the RPKM threshold < 5 in all conditions are not shown. These are *iha*, *tccP*, *espK*, *espX1*, *espX2*, *espR2’*, *espL1*, *espY4*, *espY5’*, *espL4*, *espX5*, *lpfCC’*, *lpfB*. Note that most genes have highest RPKM values in LB.

| gene tag | product | LB | LB-pH9 |  | LB-pH4 |  | LB-15°C |  | LB-nitrite |  | LB-antibiotics |  | LB-solid |  | minimal medium |  | spinach juice |  | radish sprouts |  | faeces |  |
| --- | --- | --- | --- | --- | --- | --- | --- | --- | --- | --- | --- | --- | --- | --- | --- | --- | --- | --- | --- | --- | --- | --- |
| Z0065 | hypothetical protein Z0065, espY1 | 1 (23) | 0,3 (16) |  | -0,1 (3) |  | -0,3 (15) |  | 0,3 (26) |  | 0,9 (11) |  | 0,1 (8) |  | -1,9 (4) |  | -1,8 (4) |  | -0,7 (8) |  | -7,0 (0) |  |
| Z0078 | hypothetical protein Z0078, espY2 | 1 (91) | 0,9 (97) |  | -0,5 (10) |  | 0,2 (85) |  | 0,8 (144) |  | **-3,0 (3)** |  | 1,1 (63) |  | -0,4 (41) |  | -0,3 (41) |  | -0,8 (30) |  | 1,4 (38) |  |
| Z0521 | hypothetical protein Z0521, espY3 | 1 (7) | -0,8 (3) |  | -1,2 (1) |  | -0,6 (4) |  | -0,7 (4) |  | 0,7 (3) |  | 1,2 (6) |  | -0,4 (3) |  | 0,8 (8) |  | -1,6 (1) |  | 1,6 (4) |  |
| Z0841 | DNA-binding transcriptional activator KdpE | 1 (11) | -1,6 (2) |  | -0,5 (1) |  | -1,5 (3) |  | -1,2 (4) |  | -5,8 (0) |  | -0,1 (3) |  | **-5,8 (0)** |  | 0,2 (7) |  | 0,5 (9) |  | 1,0 (4) |  |
| Z0985 | hypothetical protein Z0985, nleB2-1 | 1 (30) | -1,1 (9) |  | -1,6 (2) |  | **-2,1 (6)** |  | -1,8 (8) |  | 1,3 (20) |  | **3,0 (81)** |  | -2,0 (5) |  | 0,8 (30) |  | **-1,7 (5)** |  | **3,5 (60)** |  |
| Z0986 | hypothetical protein Z0986, nleC | 1 (25) | -1,2 (7) |  | 0,0 (4) |  | -1,3 (9) |  | -1,3 (10) |  | 1,5 (18) |  | 0,3 (11) |  | -1,1 (7) |  | 0,0 (14) |  | **-1,8 (4)** |  | -0,2 (4) |  |
| Z0989 | hypothetical protein Z0989, nleH1-1 | 1 (11) | -0,4 (5) |  | -6,1 (0) |  | -0,4 (6) |  | **-2,5 (2)** |  | 1,1 (6) |  | -0,4 (3) |  | -0,6 (4) |  | -1,3 (2) |  | -1,2 (2) |  | 1,3 (4) |  |
| Z0990 | hypothetical protein Z0990, nleD | 1 (44) | 0,4 (36) |  | -2,7 (1) |  | 0,2 (46) |  | -0,3 (35) |  | **-8,0 (0)** |  | -0,7 (10) |  | 0,3 (36) |  | -1,1 (12) |  | **-1,7 (8)** |  | -2,1 (2) |  |
| Z1385 | putative secreted protein, espF2-1' | 1 (7) | 0,7 (7) |  | **2,2 (5)** |  | 1,5 (17) |  | 0,8 (12) |  | -5,4 (0) |  | 1,9 (9) |  | 0,9 (8) |  | -2,1 (1) |  | 0,0 (4) |  | 1,4 (3) |  |
| Z1387 | hypothetical protein Z1387, espV' | 1 (43) | **-3,2 (2)** |  | -0,6 (5) |  | **-3,4 (3)** |  | -1,8 (11) |  | 1,7 (36) |  | 1,7 (45) |  | **-5,9 (0)** |  | 1,5 (70) |  | -0,1 (23) |  | -5,9 (0) |  |
| Z1464 | shiga-like toxin II A subunit, stx2A | 1 (175) | -1,5 (36) |  | 0,0 (28) |  | -1,8 (42) |  | -0,2 (141) |  | 0,5 (63) |  | -0,5 (40) |  | -1,6 (35) |  | **-2,4 (18)** |  | 1,0 (203) |  | **-10,3 (0)** |  |
| Z1465 | shiga-like toxin II B subunit, stx2B | 1 (328) | -1,2 (84) |  | -0,9 (27) |  | -0,7 (167) |  | 0,5 (432) |  | 1,5 (241) |  | 1,1 (232) |  | -0,6 (130) |  | -1,5 (67) |  | 0,9 (349) |  | 1,5 (153) |  |
| Z2075 | hypothetical protein Z2075, nleG2-4' | 1 (42) | **-2,1 (6)** |  | **-7,4 (0)** |  | -1,3 (16) |  | -0,9 (22) |  | **-7,4 (0)** |  | 0,2 (17) |  | 0,0 (27) |  | **-4,1 (1)** |  | -1,6 (9) |  | 0,0 (7) |  |
| Z2077 | hypothetical protein Z2077, nleG7' | 1 (23) | -0,9 (8) |  | 1,0 (8) |  | 0,9 (39) |  | -0,6 (15) |  | 1,0 (13) |  | 1,1 (17) |  | 0,3 (18) |  | 0,4 (18) |  | -1,0 (7) |  | 1,7 (13) |  |
| Z2149 | hypothetical protein Z2149, nleG2-3 | 1 (135) | **-1,9 (21)** |  | **-2,9 (3)** |  | **-3,1 (12)** |  | **-2,8 (18)** |  | **-3,6 (3)** |  | -1,9 (12) |  | **-3,1 (9)** |  | **-3,7 (6)** |  | **-2,0 (19)** |  | **-9,2 (0)** |  |
| Z2150 | hypothetical protein Z2150, nleG6-2 | 1 (156) | **-2,0 (23)** |  | **-2,3 (5)** |  | -1,3 (52) |  | **-3,0 (17)** |  | -1,9 (10) |  | -0,1 (49) |  | **-3,2 (10)** |  | **-6,2 (1)** |  | **-3,7 (7)** |  | **-9,5 (0)** |  |
| Z2151 | hypothetical protein Z2151, nleG5-2 | 1 (59) | -1,4 (12) |  | **-8,1 (0)** |  | **-2,1 (11)** |  | **-2,3 (11)** |  | **-8,1 (0)** |  | -1,0 (9) |  | **-2,6 (6)** |  | **-8,1 (0)** |  | **-4,2 (2)** |  | **2,6 (59)** |  |
| Z2337 | hypothetical protein Z2337, nleG5-1 | 1 (27) | -0,2 (16) |  | **-7,3 (0)** |  | -0,9 (14) |  | -1,9 (7) |  | **-7,3 (0)** |  | -0,1 (9) |  | -1,2 (8) |  | **-3,0 (2)** |  | **-2,8 (2)** |  | **2,5 (28)** |  |
| Z2338 | hypothetical protein Z2338, nleG6-1 | 1 (46) | -0,8 (17) |  | -1,1 (4) |  | -0,6 (28) |  | -1,3 (20) |  | -2,3 (3) |  | -0,1 (15) |  | -1,8 (9) |  | **-3,7 (2)** |  | **-3,5 (3)** |  | **-7,9 (0)** |  |
| Z2339 | hypothetical protein Z2339, nleG2-2 | 1 (129) | -0,4 (60) |  | **-2,9 (3)** |  | -1,1 (52) |  | -1,4 (48) |  | **-3,6 (3)** |  | 0,7 (71) |  | -1,8 (24) |  | **-2,4 (15)** |  | **-1,7 (26)** |  | -2,4 (4) |  |
| Z2560 | hypothetical protein Z2560, nleG9' | 1 (6) | -0,4 (3) |  | 0,7 (2) |  | 0,2 (6) |  | -0,1 (5) |  | 1,0 (3) |  | 1,9 (7) |  | -1,0 (2) |  | **2,0 (14)** |  | **-4,6 (0)** |  | **5,0 (33)** |  |
| Z2565 | putative chaperone protein, espM1 | 1 (1514) | 0,7 (1513) |  | -0,1 (255) |  | 0,1 (1438) |  | 0,0 (1472) |  | **-2,5 (74)** |  | 0,4 (704) |  | **-2,3 (202)** |  | **-3,2 (103)** |  | **-2,5 (158)** |  | **-2,2 (58)** |  |
| Z3071 | hypothetical protein Z3071, espJ | 1 (132) | **-2,2 (18)** |  | -0,8 (13) |  | **-2,8 (16)** |  | -1,8 (37) |  | -0,2 (30) |  | -1,4 (17) |  | 0,3 (104) |  | -1,8 (23) |  | **-3,5 (6)** |  | -0,8 (13) |  |
| Z3343 | shiga-like toxin 1 subunit B, stx1B | 1 (310) | **2,5 (1196)** |  | 1,2 (134) |  | 0,3 (358) |  | 1,5 (894) |  | 1,2 (211) |  | **2,3 (575)** |  | **1,7 (715)** |  | -1,2 (87) |  | **-1,7 (57)** |  | -0,4 (45) |  |
| Z3344 | shiga-like toxin 1 subunit A, stx1A | 1 (183) | **1,6 (356)** |  | 0,9 (61) |  | 0,0 (162) |  | 0,8 (312) |  | 0,7 (81) |  | **1,9 (232)** |  | 0,8 (216) |  | -1,0 (54) |  | -1,0 (54) |  | -0,9 (17) |  |
| Z3549 | NADH dehydrogenase transcriptional regulator, lhrA | 1 (165) | 1,5 (278) |  | 1,2 (64) |  | -0,6 (91) |  | 1,0 (313) |  | -0,3 (35) |  | 0,3 (68) |  | -0,4 (81) |  | -1,4 (37) |  | -0,8 (55) |  | 0,7 (45) |  |
| Z3833 | putative 2-component sensor protein, yfhK | 1 (26) | -0,6 (10) |  | -0,5 (3) |  | 0,7 (34) |  | -0,6 (16) |  | -1,5 (2) |  | 0,6 (12) |  | -0,4 (12) |  | -0,8 (8) |  | 0,1 (16) |  | **-8,2 (0)** |  |
| Z3918 | putative chaperone protein, espM2 | 1 (85) | -0,2 (45) |  | 0,3 (17) |  | -0,3 (58) |  | 0,7 (131) |  | 0,5 (30) |  | 0,2 (32) |  | **3,7 (670)** |  | **3,3 (473)** |  | -0,7 (29) |  | 1,2 (33) |  |
| Z3919 | hypothetical protein Z3919, nleG8-2 | 1 (11) | -1,2 (3) |  | -5,8 (0) |  | -0,7 (6) |  | -0,1 (9) |  | **3,1 (25)** |  | -1,6 (1) |  | **2,9 (53)** |  | 1,7 (21) |  | -0,3 (5) |  | 1,6 (6) |  |
| Z3920 | hypothetical protein Z3920, espW | 1 (10) | -0,8 (3) |  | 1,5 (5) |  | -1,5 (3) |  | -0,7 (6) |  | **2,4 (14)** |  | 0,6 (5) |  | **1,8 (22)** |  | 0,6 (9) |  | -1,5 (2) |  | 1,0 (3) |  |
| Z3921 | hypothetical protein Z3921, nleG6-3' | 1 (66) | -1,4 (15) |  | -1,6 (3) |  | -0,9 (29) |  | -1,9 (16) |  | **-7,9 (0)** |  | -1,4 (8) |  | -2,1 (9) |  | **-2,7 (6)** |  | **-2,7 (6)** |  | -0,5 (8) |  |
| Z4049 | RNA polymerase sigma factor RpoS | 1 (56) | 1,3 (84) |  | 0,3 (12) |  | 1,0 (92) |  | **2,2 (247)** |  | **-8,7 (0)** |  | -0,1 (17) |  | 1,5 (98) |  | 0,5 (44) |  | 0,1 (35) |  | -0,9 (5) |  |
| Z4326 | putative enterotoxin, espL2 | 1 (28) | -0,3 (14) |  | -1,7 (1) |  | -0,8 (14) |  | 0,1 (29) |  | -0,6 (5) |  | 0,0 (10) |  | -0,7 (11) |  | -0,4 (13) |  | **-2,1 (4)** |  | **1,7 (16)** |  |
| Z4328 | hypothetical protein Z4328, nleB | 1 (45) | -0,9 (15) |  | -0,4 (6) |  | -0,8 (23) |  | 0,1 (46) |  | **1,7 (41)** |  | -0,7 (10) |  | 0,0 (29) |  | -0,6 (18) |  | **-1,8 (7)** |  | -1,7 (2) |  |
| Z4329 | hypothetical protein Z4329, nleE | 1 (78) | -1,4 (16) |  | **-3,3 (1)** |  | **-2,0 (16)** |  | -0,8 (42) |  | 1,5 (55) |  | -1,5 (9) |  | -0,5 (33) |  | -0,6 (28) |  | **-4,1 (2)** |  | -1,8 (4) |  |
| Z4377 | DNA-binding transcriptional regulator QseB | 1 (1) | 1,7 (2) |  | -2,5 (0) |  | 1,4 (2) |  | 1,1 (2) |  | -2,5 (0) |  | **3,2 (3)** |  | 1,1 (1) |  | **4,2 (12)** |  | 1,9 (2) |  | 3,3 (2) |  |
| Z4378 | sensor protein QseC | 1 (3) | 0,8 (3) |  | -5,1 (0) |  | 0,2 (3) |  | 0,7 (5) |  | **2,5 (5)** |  | **2,2 (5)** |  | 1,0 (4) |  | **2,3 (9)** |  | 0,3 (2) |  | 1,7 (2) |  |
| Z4911 | outer membrane heme/hemoglobin receptor, chuA | 1 (3) | -0,1 (2) |  | -0,4 (0) |  | -0,8 (2) |  | **3,2 (30)** |  | -5,7 (0) |  | -0,5 (1) |  | **7,6 (402)** |  | -0,5 (1) |  | 0,0 (2) |  | -5,7 (0) |  |
| Z4965 | putative fimbrial subunit, lpfE | 1 (5) | 1,0 (7) |  | 1,7 (3) |  | **2,2 (23)** |  | 1,3 (13) |  | -4,6 (0) |  | -4,6 (0) |  | -4,6 (0) |  | 0,6 (5) |  | -0,7 (2) |  | 1,3 (2) |  |
| Z4966 | putative fimbrial protein, lpfD | 1 (18) | 0,4 (13) |  | -1,8 (1) |  | 0,1 (16) |  | 0,7 (27) |  | **-7,1 (0)** |  | -0,4 (4) |  | -1,3 (4) |  | 0,0 (10) |  | -1,1 (5) |  | 0,7 (5) |  |
| Z4971 | putative major fimbrial subunit, lpfA | 1 (5) | -0,7 (2) |  | 1,0 (2) |  | 1,5 (11) |  | -0,7 (3) |  | 1,3 (3) |  | -0,1 (1) |  | 0,8 (5) |  | 1,4 (8) |  | **-4,4 (0)** |  | 2,5 (5) |  |
| Z5100 | hypothetical protein Z5100, espF1 | 1 (20) | **-6,8 (0)** |  | -1,5 (1) |  | **-6,8 (0)** |  | **-3,2 (2)** |  | 1,8 (17) |  | **-6,8 (0)** |  | -1,0 (6) |  | **-3,5 (1)** |  | **-6,8 (0)** |  | -6,8 (0) |  |
| Z5636 | hypothetical protein Z5636, espX4 | 1 (11) | **-2,2 (1)** |  | -0,8 (1) |  | 1,3 (24) |  | **-4,4 (0)** |  | -1,5 (1) |  | -1,4 (1) |  | **-7,1 (0)** |  | -1,6 (2) |  | **-2,3 (1)** |  | -7,1 (0) |  |
| Z5935 | hypothetical protein Z5935, espX6 | 1 (4) | 0,5 (3) |  | 0,3 (1) |  | 1,4 (9) |  | 1,5 (10) |  | **2,4 (5)** |  | -0,9 (1) |  | -0,9 (1) |  | 1,2 (5) |  | -0,9 (1) |  | -0,2 (1) |  |
| Z6010 | hypothetical protein Z6010, nleG | 1 (260) | -0,8 (80) |  | 0,3 (47) |  | **-1,9 (52)** |  | **-1,9 (58)** |  | 0,1 (68) |  | 0,5 (107) |  | -1,6 (47) |  | -1,8 (40) |  | **-1,7 (42)** |  | 1,1 (86) |  |
| Z6020 | hypothetical protein Z6020, nleF | 1 (216) | -0,5 (90) |  | -1,3 (14) |  | 0,6 (271) |  | -1,3 (82) |  | **-2,7 (9)** |  | 0,4 (90) |  | -0,4 (98) |  | -0,9 (65) |  | **-2,2 (27)** |  | 0,1 (38) |  |
| Z6021 | hypothetical protein Z6021, nleH1-2 | 1 (149) | -0,3 (74) |  | **-2,5 (5)** |  | 0,3 (156) |  | -1,1 (68) |  | -1,9 (11) |  | 0,1 (56) |  | -0,9 (51) |  | -1,2 (39) |  | **-2,4 (17)** |  | -0,2 (23) |  |
| Z6024 | hypothetical protein Z6024, nleA | 1 (92) | -0,5 (41) |  | **-2,1 (4)** |  | 0,1 (92) |  | 0,1 (104) |  | -0,7 (16) |  | **-2,9 (5)** |  | 1,3 (150) |  | -1,8 (17) |  | **-1,9 (14)** |  | 0,0 (17) |  |
| Z6025 | hypothetical protein Z6025, nleG2-1' | 1 (33) | -0,6 (14) |  | -0,3 (5) |  | 0,8 (52) |  | -0,4 (26) |  | **-7,2 (0)** |  | -0,7 (7) |  | -0,2 (19) |  | -1,7 (6) |  | -1,7 (6) |  | -0,4 (5) |  |
